# A genome‐scale TF–DNA interaction network of transcriptional regulation of *Arabidopsis* primary and specialized metabolism

**DOI:** 10.15252/msb.202110625

**Published:** 2021-11-24

**Authors:** Michelle Tang, Baohua Li, Xue Zhou, Tayah Bolt, Jia Jie Li, Neiman Cruz, Allison Gaudinier, Richard Ngo, Caitlin Clark‐Wiest, Daniel J Kliebenstein, Siobhan M Brady

**Affiliations:** ^1^ Department of Plant Biology and Genome Center University of California, Davis Davis CA USA; ^2^ Department of Plant Sciences University of California, Davis Davis CA USA; ^3^ Plant Biology Graduate Group University of California, Davis Davis CA USA; ^4^ DynaMo Center of Excellence University of Copenhagen Frederiksberg C Denmark

**Keywords:** CCPs central carbon promoters, GSL glucosinolate, TF transcription factor, Y1H yeast one‐hybrid, Chromatin, Transcription & Genomics, Metabolism, Plant Biology

## Abstract

Plant metabolism is more complex relative to individual microbes. In single‐celled microbes, transcriptional regulation by single transcription factors (TFs) is sufficient to shift primary metabolism. Corresponding genome‐level transcriptional regulatory maps of metabolism reveal the underlying design principles responsible for these shifts as a model in which master regulators largely coordinate specific metabolic pathways. Plant primary and specialized metabolism occur within innumerable cell types, and their reactions shift depending on internal and external cues. Given the importance of plants and their metabolites in providing humanity with food, fiber, and medicine, we set out to develop a genome‐scale transcriptional regulatory map of *Arabidopsis* metabolic genes. A comprehensive set of protein–DNA interactions between *Arabidopsis thaliana* TFs and gene promoters in primary and specialized metabolic pathways were mapped. To demonstrate the utility of this resource, we identified and functionally validated regulators of the tricarboxylic acid (TCA) cycle. The resulting network suggests that plant metabolic design principles are distinct from those of microbes. Instead, metabolism appears to be transcriptionally coordinated via developmental‐ and stress‐conditional processes that can coordinate across primary and specialized metabolism. These data represent the most comprehensive resource of interactions between TFs and metabolic genes in plants.

## Graphical abstract

A transcription factor‐promoter interaction network of primary and specialized metabolism in *Arabidopsis thaliana* reveals transcriptional coordination across multiple metabolic pathways that is developmental and stress condition‐dependent.

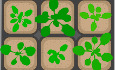

## Introduction

Metabolism is the fundamental biological process underpinning all cellular functions. An organism’s metabolism comprises individual biochemical reactions organized into metabolic pathways where metabolites are sequentially transformed in increasing or decreasing complexity by enzymes. In conjunction, the primary metabolic pathways create the cellular building blocks and directly contribute to the interconversion of chemicals into energy currency. In plants, and most other organisms, primary metabolites serve as precursors to secondary, or specialized, metabolites crucial to the organism’s interaction with its environment. Plant specialized metabolites serve many functions, including defending plants from predators and pathogens, attracting symbiotic organisms, and promoting interactions with pollinators.

To properly function, metabolic pathways must be intricately orchestrated to maintain the homeostasis necessary for growth and are in turn dependent on the organism’s developmental stage and environment. Thus, it is critical to understand how metabolic pathways are regulated to maximize our ability to predict and manipulate an organism’s genotype‐to‐phenotype matrix. Metabolism is known to be regulated by mechanisms that span the central dogma from messenger RNA (mRNA) transcription to protein posttranslational modifications, with the best‐studied regulatory mechanisms being posttranslational modification and allosteric feedback of enzymes (Nielsen, 2017). Adding to this understanding, systems biology and genetic approaches in single‐celled organisms demonstrate the importance of transcriptional regulation. In *Saccharomyces cerevisiae* and *Escherichia coli*, these systems approaches integrate chromatin immunoprecipitation, transcriptomic experiments, and *in silico* models to elucidate transcription factor (TF)–enzyme promoter regulatory interactions and ultimately, investigation of genome‐scale regulatory networks of global metabolism (Ihmels *et al*, 2002, 2004; Barrett *et al*, 2005; Fang *et al*, 2017; Lempp *et al*, 2019). Studies in these organisms have resulted in a model where metabolic networks are organized into distinct transcriptional modules that control specific cellular processes (Ihmels *et al*, 2002, 2004). Whether the same principles apply in multicellular organisms remains to be determined.

Relative to *S. cerevisiae* and *E. coli*, the genomes of multicellular organisms encode many more genes including TFs, enzymes and in plants, relative to animals, a further expansion of both enzyme and TF families linked to metabolism. The acquisition of multicellularity also enables the partitioning of function across cell types. Thus, multicellular organisms likely have more complex transcriptional and metabolic regulation in comparison with single‐celled microbes. However, few studies exist that systematically characterize the complexity of metabolic networks from the perspective of transcriptional regulation in multicellular organisms. Even fewer studies have explored the regulatory interconnection between the regulation of central and specialized metabolism in multicellular organisms, where specialized metabolism is a critical component of the organism’s response to the environment. Instead, the majority of studies on plant metabolism have focused on the transcriptional regulation of individual pathways (Bonawitz *et al*, 2012; Li *et al*, 2014, 2018; Kim *et al*, 2015; Dolan *et al*, 2017; Gaudinier *et al*, 2018).

Recent work is beginning to show that plant primary and specialized metabolism are highly interconnected. The potential for interconnected regulation of central carbon and specialized metabolism is revealed by transcriptional profiling of mutants in TFs, MYB28 and MYB29, that regulate glucosinolate (GSL) biosynthesis, a specialized metabolic pathway. This work showed that, as expected, these TFs not only regulate the GSL biosynthetic pathway, but also affect several primary metabolic pathways that synthesize precursors of GSLs, including methionine biosynthesis, tryptophan biosynthesis, the TCA cycle, sulfur metabolism, and folate metabolism (Malitsky *et al*, 2008; Sønderby *et al*, 2010). Another example of TFs that affect both central carbon and specialized metabolism are the Mediator complex, a multisubunit transcriptional co‐regulator that interacts with other TFs, and the general transcription machinery to regulate transcription (Tsai *et al*, 2014). In *Arabidopsis*, different subunits of the Mediator complex are involved in fatty acid biosynthesis (Kim *et al*, 2016) and the synthesis of specialized metabolites known as phenylpropanoids (Stout *et al*, 2008; Bonawitz *et al*, 2012; Dolan *et al*, 2017; Dolan & Chapple, 2018). These observations suggest that transcriptional regulation of plant metabolism may be highly coordinated and differs from the pathway‐specific transcriptional regulatory modules, as observed in *S. cerevisiae* and *E. coli*. Thus, we hypothesized that *Arabidopsi*s metabolism is orchestrated by TFs that regulate both primary and specialized metabolism.

Testing this hypothesis requires a large‐scale dataset linking as many TFs to primary metabolism promoters as possible. Presently, large‐scale regulatory transcriptional regulatory networks of plant metabolism can be inferred computationally with tools and publicly available datasets including RNA sequencing (RNA‐Seq), chromatin immunoprecipitation sequencing (ChIP‐Seq), DNA affinity purification sequencing (DAP‐Seq), and protein‐binding microarrays (Weirauch *et al*, 2014; O'Malley *et al*, 2016; Kulkarni *et al*, 2018). However, these datasets are limited to a subset of TFs, with the *Arabidopsis* cistrome dataset consisting of 529 of 2,492 *Arabidopsis* TFs predicted to be in the genome (Pruneda‐Paz *et al*, 2014; O'Malley *et al*, 2016). We complement these approaches using enhanced yeast one‐hybrid (Y1H) assays (Gaudinier *et al*, 2011; Reece‐Hoyes *et al*, 2011a, b) to systematically screen for interactions between approximately 85% of all characterized and putative TFs in *Arabidopsis* and 224 promoters of enzyme genes in 11 central carbon metabolic pathways and of a specialized metabolic pathway, aliphatic GSL biosynthesis. By developing a comprehensive dataset linking TFs to metabolic genes, we can begin addressing key questions on the transcriptional regulation of multicellular metabolism: are metabolic pathways linked by common precursors coordinately regulated by single TFs? Do TFs confer environmental conditionality of primary metabolism as occurs for specialized metabolism?

This dataset showed that 90% of TFs bind to promoters of metabolic genes, with all TFs binding to gene promoters in multiple metabolic pathways. Co‐expression analyses support a regulatory model whereby the majority of TFs influence more than one metabolic pathway. We validated this hypothesis and the dataset by expression profiling inducible lines of four TFs that bind to promoters of genes from a range of metabolic pathways. This showed that the predicted target enzyme genes in the central carbon metabolic pathways were enriched among the differentially expressed genes (DEGs) of the TFs we examined. Additionally, we explored the combinatorial regulation of a single pathway, the TCA cycle, given its interconnection with other central metabolic pathways. This dataset provides a unique resource to extend our understanding of how metabolism is regulated in multicellular organisms.

## Results

### Genome‐scale identification of TFs involved in primary and secondary metabolism

**Figure 1:**
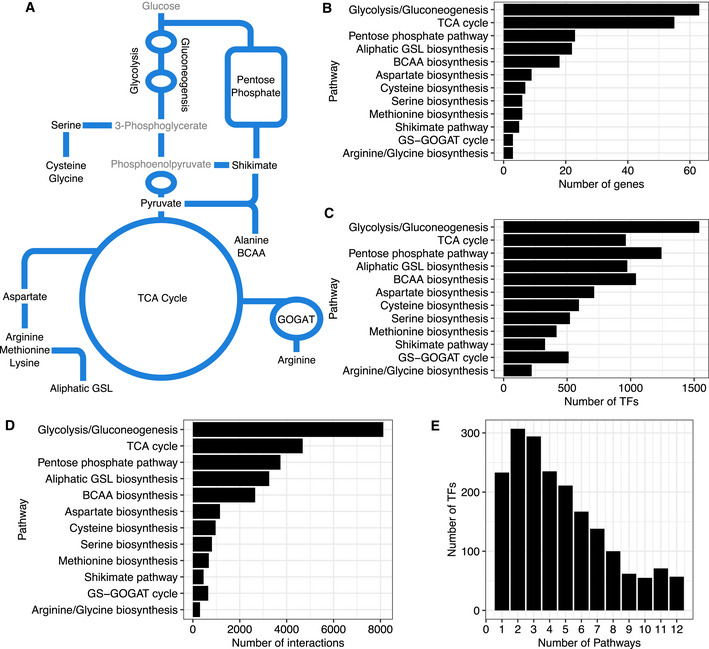
Summary of transcription factor (TF)–promoter interactions of central carbon and specialized metabolism AThe simplified biochemical network comprises amino acid biosynthetic and respiratory pathways in central carbon metabolism and a specialized metabolic pathway, aliphatic glucosinolate (GSL) biosynthesis, studied in this paper.BEach pathway in central carbon and specialized metabolism varies in gene number.C, DYeast one‐hybrid (Y1H) identified TFs that interact with promoters of genes in each pathway in central carbon and in the specialized metabolic pathway.EThe majority of TFs bind to promoters from two or more metabolic pathways.

To identify TFs that potentially regulate *Arabidopsis* primary metabolism, we conducted enhanced Y1H screens (Gaudinier *et al*, 2011; Reece‐Hoyes *et al*, 2011a, b) with 224 promoters of enzyme‐encoding genes involved in the TCA cycle, glycolysis and gluconeogenesis, pentose phosphate pathway, glutamine synthetase/glutamine oxoglutarate aminotransferase (GS–GOGAT) cycle, shikimate pathway, and most amino acid biosynthesis pathways, with the exception of the aromatic amino acids (Fig 1A, Dataset EV1). We refer to this collective group of promoters as central carbon promoters (CCPs). We also included promoters of genes involved in aliphatic GSL biosynthesis (Li *et al*, 2014), to investigate transcriptional coordination of primary and secondary metabolism. To enable the genome‐level detection of TFs involved in primary metabolism, we extended our original collection of 812 TFs by cloning an additional 1,224 TFs into activation domain fusion vectors compatible with our enhanced Y1H system (Dataset EV2; Gaudinier *et al,* 2011, 2018; Li *et al,* 2014). The final *Arabidopsis* TF collection contains 2,039 TFs and represents over 80% of all characterized and putative TFs in *Arabidopsis* (Pruneda‐Paz *et al*, 2014; see https://genomecenter.ucdavis.edu/yeast‐one‐hybrid‐services).

**Figure 2:**
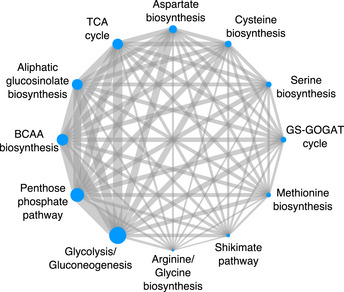
Pairwise association of transcription factors (TFs) between metabolic pathways The number of TFs shared between metabolic pathways is greater than expected by chance for all combinations of pairs of metabolic pathways (Dataset EV4). The size of the nodes corresponds to the number of TFs identified for each pathway. The width of the edge linking two metabolic pathways indicates the number of TFs shared between the two pathways.

From the binding data obtained via the genome‐scale Y1H, we queried how promoters and metabolic pathways are organized. We detected 27,485 interactions between 1,930 TFs and 220 promoters across the 12 pathways surveyed (Figs 1A–C, Dataset EV3). Per promoter, we identified 1–509 TF interactions, with an average of 125 TFs binding to a promoter. To visualize if most TFs are specific to individual pathways or connect to multiple metabolic pathways, we mapped TF–promoter interactions relative to their respective pathways (Fig 1B and C). While we observed a general positive relationship between the number of interactions and the number of promoters screened for each pathway (Fig 1B and D), the number of TFs identified did not scale with the number of promoters in each pathway. For instance, we detected approximately the same number of TFs binding to promoters associated with serine biosynthesis and the GOGAT cycle, even though there are twice as many genes encoding enzymes in serine biosynthesis as in the GOGAT cycle. While the large number of TFs found to bind to metabolic promoters could suggest that specific groups of TFs target unique pathways, the majority of TFs (˜88%) in our network bind to gene promoters in two or more pathways (Fig 1E). Indeed, only 10% of the TFs bound to promoters of genes in a single pathway. This pattern of binding suggests TFs may regulate multiple pathways to coordinate metabolic control (Fig 1E). We next queried the percentage of TFs that are shared between metabolic pathways. In all pairwise combinations, the number of TFs common between any two pathways is significantly greater than expected (Fisher’s exact test, false discovery rate (FDR) < 0.0001), signifying that transcriptionally mediated interconnection may be an emergent property of plant metabolism (Fig 2, Dataset EV4). Such coordination may be guided to control a particular biological process or molecular function that requires a subset of metabolic pathways. However, Gene Ontology (GO) enrichment analysis for each pathway did not provide any clarity, likely due to the fact that most TFs do not have known functions or metabolism‐associated annotations (Dataset EV5).

We next examined how our representative specialized metabolic pathway, aliphatic GSL biosynthesis, is integrated with central carbon metabolism within the TF–DNA interaction network. Among the 974 TFs that bind to aliphatic GSL gene promoters, 933 of those TFs (˜95%) also bind to promoters from a central carbon pathway. In our pairwise comparison of pathways based on TF binding, the central carbon metabolic pathways that shared the most TFs with aliphatic GSL based on the level of enrichment using a hypergeometric test are the pathways that synthesize the sulfur‐containing precursors needed to synthesize aliphatic GSL (methionine and cysteine) (Dataset EV4). This signifies that although the number of TFs shared between pathways is higher than expected across all pairwise combination of pathways, there is still regional enrichment wherein biosynthetic pathways that are closest within the metabolic network share more TFs than biosynthetic pathways more distant in the metabolic network.

### Coordinate transcriptional regulation of central carbon metabolism

**Figure 3:**
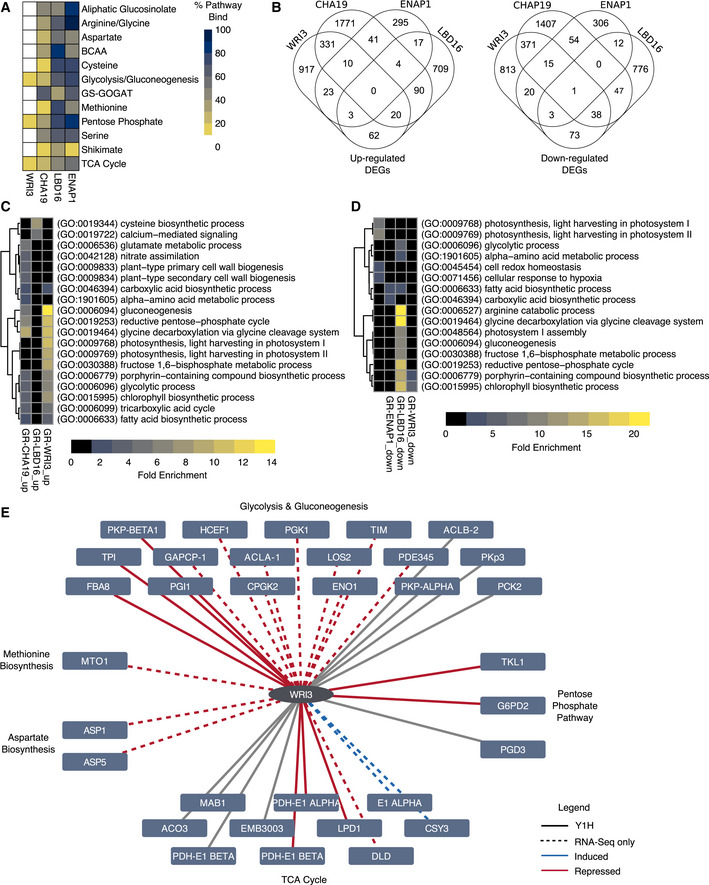
Validation of regulatory interactions via transcriptomics of glucocorticoid receptor–transcription factors (GR–TFs) Chromatin Remodeling 1 (CHA19), EIN2 Nuclear Associated Protein 1 (ENAP1), Lateral Organ Boundaries‐Domain 16 (LBD16), and Wrinkled 3 (WRI3) vary in the pathways and the number of genes targeted based on yeast one‐hybrid (Y1H).Thousands of genes were differentially expressed in dexamethasone (Dex)‐induced GR–TFs compared to GR–TFs under mock conditions.Gene ontologies (GOs) significantly enriched in the upregulated differentially expressed genes (DEGs) included metabolic pathways in central carbon metabolism.Gene ontologies (GOs) significantly enriched in the downregulated DEGs related to central carbon metabolism were found mainly in glucocorticoid‐Lateral Organ Boundaries‐Domain 16 (GR‐LBD16).Wrinkled 3 (WRI3) was enriched for targets in glycolysis/gluconeogenesis and the tricarboxylic acid (TCA) cycle. The WRI3 network consists of interactions identified in the Y1H and through expression profiling of GR‐WRI3. Solid gray lines indicate WRI3–target interactions found in the Y1H network only. Dashed lines signify interactions detected by RNA‐Seq only. Colored solid lines indicate interactions identified via Y1H and RNA‐Seq. Lines are colored by whether GR‐WRI3 upregulates (blue) or downregulates (red) target gene expressions.

This global analysis of TF–metabolic promoter interactions suggests coordinate transcriptional regulation of central carbon and specialized metabolic pathways. However, genome‐scale assays like the Y1H suffer from both false positives and false negatives. We therefore tested the regulatory capacity of four TFs that bind to gene promoters from multiple metabolic pathways: *CHROMATIN REMODELING 19 (CHA19)*, *EIN2 NUCLEAR ASSOCIATED PROTEIN 1 (ENAP1)*, *LATERAL ORGAN BOUNDARIES‐DOMAIN 16 (LBD16)*, and *WRINKLED 3 (WRI3)* (Table 1). WRI3 binds to promoters in the TCA cycle, glycolysis/gluconeogenesis and pentose phosphate pathway, while CHA19, ENAP1, and LBD16 bind to promoters from all 12 pathways at varying degrees (Table 1, Fig 3A). We predicted that conditional overexpression of these TFs would reveal their sufficiency to regulate gene expression in these pathways. Additionally, we utilized the fact that dark‐grown plant seedlings are heterotrophic and do not shift to autotrophy (photosynthesis) until exposure to light. Thus, we tested the regulatory capacity for these TFs in dark‐grown seedlings. We reasoned that carbon metabolism relating to photosynthesis and carbon fixation would be suppressed in dark‐grown seedlings, and that the seedlings would maintain heterotrophy during catabolism of maternal stores to provide energy for seed germination and growth. *CHA19*, *ENAP1*, *LBD16*, and *WRI3* coding regions were fused to a dexamethasone (Dex)‐controlled glucocorticoid receptor (GR). The gene expression was profiled by RNA‐sequencing 24 h after mock treatment or 10 μM dexamethasone induction in dark‐grown six‐day‐old *Arabidopsis* seedlings. Hundreds to thousands of genes were differentially expressed in response to TF induction across multiple insertion lines (Fig 3B, Dataset EV6). There was a significant enrichment of Y1H network gene targets among these differentially expressed genes (DEGs) for *GR‐ENAP1; GR‐LBD16*, and *GR‐WRI3* (one‐tailed Fisher’s exact test, *P* < 0.05).

**Table 1:** Associated metabolic pathways of promoters bound by transcription factors tested in conditional glucocorticoid receptor (GR)‐induction assays.

	CHA19	ENA1	LBD16	WRI3
Aliphatic GSL biosynthesis (22)	9	18	9	0
Arginine/glycine biosynthesis (3)	1	3	2	0
Aspartate biosynthesis (9)	2	4	5	0
BCAA biosynthesis (18)	6	8	15	0
Cysteine biosynthesis (8)	1	6	6	0
Glycolysis/gluconeogenesis (64)	19	49	41	8
GS‐GOGAT cycle (3)	2	2	1	0
Methionine biosynthesis (7)	1	3	5	0
Pentose phosphate pathway (25)	8	21	20	3
Serine biosynthesis (6)	3	4	4	0
Shikimate pathway (5)	1	1	2	0
TCA cycle (56)	15	33	24	7

BCAA, branched‐chain amino acid; GS‐GOGAT, glutamine synthetase/glutamine oxoglutarate aminotransferase; GSL, glucosinolate; TCA, tricarboxylic acid.

Summary of the number of targets in each metabolic pathway of Chromatin Remodeling 19 (CHA19), EIN2 Nuclear‐Associated Protein 1 (ENAP1), Lateral Organ Boundaries‐Domain 16 (LBD16), and Wrinkled 3 (WRI3) from yeast one‐hybrid (Y1H) assays. The number of promoters of each metabolic pathway assayed is given in parentheses.

Gene ontology and pathway enrichment analysis further corroborated the effect of these TFs on central carbon metabolism. Gene ontologies (GOs) associated with glycolysis/gluconeogenesis, glycine catabolism, and the TCA cycle were overrepresented more than expected by chance in the significantly upregulated DEGs upon GR induction of *CHA19* and *WRI3* (Fig 3C). Cysteine biosynthesis was enriched in significantly upregulated DEGs upon GR induction of *LBD16*. Only *GR‐LBD16* had downregulated DEGs enriched for GO terms involving central carbon metabolism (glycine and arginine catabolism, reductive pentose phosphate pathway, glycolysis, and gluconeogenesis) (Fig 3D). Pathway enrichment analysis (Methods) revealed, in particular, significant enrichment of DEGs in the TCA cycle for *GR‐WRI3*. As much as 71.4% of the Y1H‐predicted *WRI3* TCA cycle targets were recovered in the RNA‐Seq analysis (Fig 3E, *P* = 0.0006, Fisher’s exact test). Additionally, we found other TCA cycle genes, totaling 28.5% of TCA cycle genes, to be enriched in the set of significant Dex‐dependent genes (Fig 3E, *P* = 0.0018, Fisher’s exact test). These data collectively demonstrate that our TF–DNA interaction network was able to predict *in planta* regulatory interactions, in line with mapped networks for other biological processes (Li *et al*, 2014; Taylor‐Teeples *et al*, 2014; Gaudinier *et al*, 2018; Truskina *et al*, 2021).

**Figure 4:**
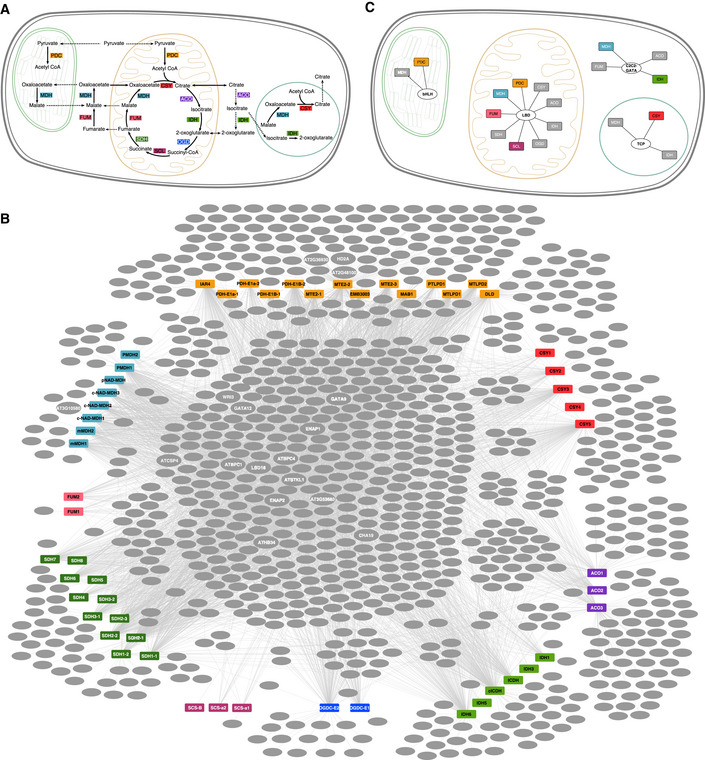
*Arabidopsis* tricarboxylic acid (TCA) cycle and its yeast one‐hybrid (Y1H) network TCA cycle‐associated metabolites and isozymes in the plastid (green), mitochondrion (orange), peroxisome (blue), and cytosol of a plant cell allow for noncyclic flux, thus increasing the flexibility of the pathway. Organelles are not drawn to scale.Y1H network shows the interactions between TFs and promoters of TCA cycle genes. Promoters are colored rectangles. The following colors correspond with the metabolic pathways: Orange PDC, pyruvate dehydrogenase; red CSY, citrate synthase; purple ACO, aconitase; light green IDH, isocitrate dehydrogenase; blue OGD, oxoglutarate dehydrogenase; light purple SCL, succinyl‐CoA ligase; green SDH, succinate dehydrogenase; pink FUM, fumarase; and light blue MDH, malate dehydrogenase. Gray ovals denote TF, and gray edges indicate interactions detected via Y1H. Tested TFs are labeled. See Fig EV1 for full diagram.TF family (oval) enrichment is modularly organized by the interaction of cellular localization and enzyme (rectangles). Colored enzymes indicate significant TF family enrichment in cellular compartment (adjusted *P* < 0.05, Fisher’s exact test).

In most heterotrophic and autotrophic eukaryotes, the TCA cycle occurs predominantly in the mitochondria. Plants, however, have expanded this repertoire such that TCA cycle isoforms also function in the cytosol, peroxisomes, and plastids, thus allowing pathway interactions with photosynthesis, photorespiration, and nitrogen assimilation. These isoforms create the potential for localized bypasses, resulting in cyclic and noncyclic fluxes in the TCA cycle to optimize metabolism (Tcherkez *et al*, 2009; Araújo *et al*, 2012). Given the relatively large number of TCA cycle genes differentially expressed upon GR induction of *WRI3*, we inspected their subcellular localization. The TCA cycle DEGs of GR‐WRI3 are specifically localized in the mitochondria and plastids (Fig 4A). This showed that only the mitochondrial and plastidic TCA pathways were being influenced by WRI3 indicating the potential for TFs to differentially modulate pathways targeted to different subcellular compartments (Fig 4A, *P* = 0.01159 (mitochondria) and *P* = 0.001431 (plastids), respectively, two‐sided Fisher’s exact test, Methods). Overall, the inducible constructs support the Y1H‐binding results and the hypothesis of coordinated regulation across metabolic pathways.

**Figure EV1:**
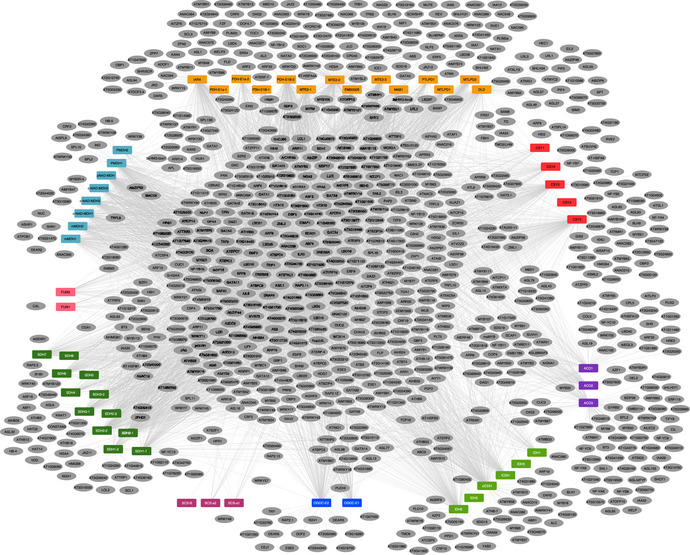
Transcription factor–tricarboxylic acid (TF–TCA) cycle target gene interaction network Yeast one‐hybrid (Y1H) assays revealed a large and combinatorial network of TF–TCA cycle target gene interactions. Colored rectangles, promoters; gray oval, transcription factor; gray edge, interaction. Orange PDC, pyruvate dehydrogenase; red CSY, citrate synthase; purple ACO, aconitase; light green IDH, isocitrate dehydrogenase; blue OGD, oxoglutarate dehydrogenase; light purple SCL, succinyl CoA ligase; green SDH, succinate dehydrogenase; pink FUM, fumarase; light blue MDH, malate dehydrogenase.

### Uncovering regulators of the *Arabidopsis* TCA cycle

To develop a deeper understanding of a single metabolic pathway, we focused on the TCA cycle that converts nutrients into carbon skeletons for more complex biomolecules and reduces electron carriers for adenosine triphosphate (ATP) synthesis. These functions are carried out by eight enzymes: citrate synthase (CSY), aconitase (ACO), isocitrate dehydrogenase (IDH), oxoglutarate dehydrogenase (OGD), succinyl‐CoA (coenzyme A) ligase (SCL), succinate dehydrogenase (SDH), fumarase (FUM), and malate dehydrogenase (MDH), many of whom have organelle‐specific isoforms (Fig 4A). Pyruvate dehydrogenase (PDH) serves as a critical link between glycolysis and the TCA cycle, which together, form the central hub of carbon metabolism. Thus, the plant TCA cycle comprises organelle‐specific isoforms, connects with many metabolic processes, and its activity likely functions in different ways in cells, tissues, organs, and the plant’s response to the environment. We therefore use the TCA cycle as a reference point to examine how TFs function to organize the TCA cycle and ultimately how this regulation coordinates plant growth, development, and response to the environment.

**Figure EV2:**
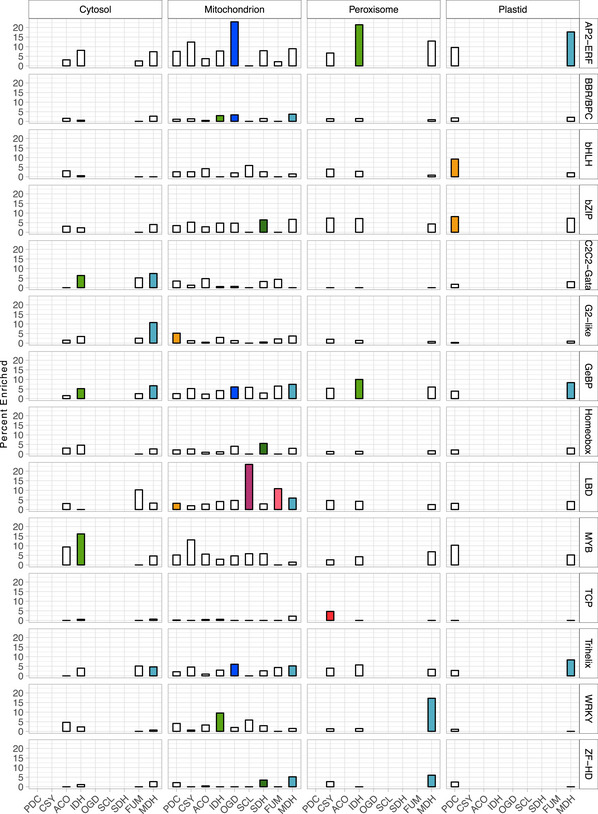
Transcription factor (TF) families are enriched for tricarboxylic acid (TCA) cycle gene targets in specific cellular compartments Bar graphs display the percentage of TFs enriched for binding to promoters of the TCA cycle enzyme in the cytosol, mitochondrion, peroxisome, and plastid. The bar is colored if the TF family targeting the TCA cycle enzyme in the cellular compartment is significant (adjusted *P* < 0.05, Fisher’s Exact Test, Dataset EV7). The colors of bars correspond to the TCA cycle enzyme in Figs 4 and EV1.

We first mapped a TCA cycle subnetwork consisting of 4,684 interactions between 962 TFs and 55 TCA cycle genes (Figs 4B and EV1, Datasets EV1 and EV3). Within this subnetwork, we tested the hypothesis that certain TF families coordinate specific isoforms within specific cellular compartments. Enrichment was constrained to specific enzyme isoforms targeted to defined cellular compartments and not for all TCA cycle enzymes in that compartment (Fig EV2). For example, TFs in the LATERAL ORGAN BOUNDARIES DOMAIN (LBD) family were enriched for binding to promoters of genes encoding the mitochondrial‐localized forms of pyruvate dehydrogenase (PDH), succinyl‐CoA ligase (SCL), fumarase (FUM), and malate dehydrogenase (MDH) (Figs 4C and EV2, Dataset EV7). These data suggest that the transcriptional control of the TCA cycle is organized around the intersection of the enzyme complex and subcellular compartment.

**Figure EV3:**
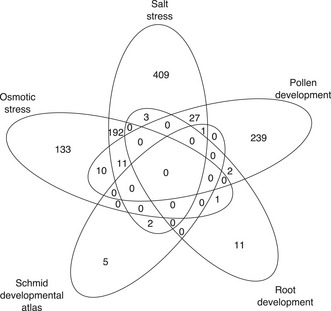
Highly correlated transcription factor–tricarboxylic acid (TF–TCA) cycle target gene interactions shared between microarray datasets Numbers in the Venn diagram represent the TF–TCA cycle target gene co‐expression with the absolute value of Pearson correlation coefficient ≥ 0.8 (Dataset EV8).

As metabolic priorities differ between plant cell types and shift while adapting to environmental changes, we predicted that a given TF’s regulation of TCA cycle genes will be structured by development and the environment. To test this prediction of context dependency, we estimated the Pearson correlation coefficients of TFs and their TCA cycle targets across five microarray datasets that surveyed different developmental processes and environments: (i) plant development (Schmid *et al*, 2005); (ii) root cell type development (Birnbaum *et al*, 2003; Lee *et al*, 2006; Levesque *et al*, 2006; Brady *et al*, 2007); (iii) pollen development (Honys & Twell, 2004; Qin *et al*, 2009); (iv) osmotic stress (Kilian *et al*, 2007); and (v) salt stress (Kilian *et al*, 2007) (Dataset EV8, Reagents Table). If our hypothesis of conditional regulation were true, we expected to detect few interactions in common across the five microarray datasets. As many as 1,046 TF/TCA cycle target interactions from the TCA cycle subnetwork (˜26%) were highly correlated (|*r*| > 0.8) in one or more microarray datasets (Fig EV3). Of the TF/TCA cycle target interactions that are highly co‐expressed, 797 interactions (˜76%) were exclusive to only one of the five microarray datasets. Roughly 23% (237) of the highly correlated interactions between TF and TCA cycle targets were found in two microarray datasets, with the vast majority of these being an overlap between related salt and osmotic stress microarrays. The absence of universal TF/TCA cycle target interaction and the high percentage of interactions being specific to a developmental or stress dataset align with our hypothesis that regulation of TCA cycle genes is highly conditional.

### Seventeen TFs contribute to TCA‐cycle‐dependent plant growth

**Figure 5:**
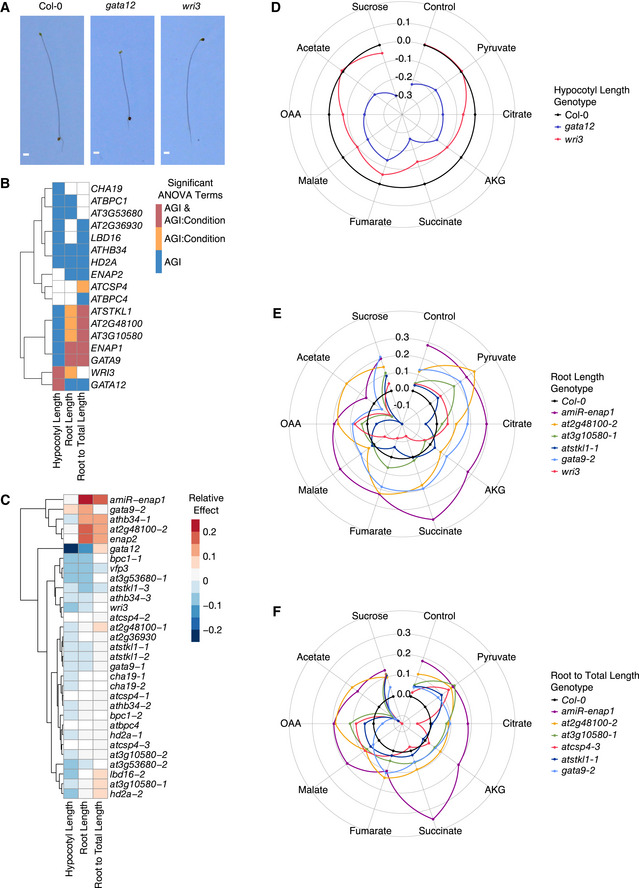
Characterization of tricarboxylic acid (TCA) cycle function in transcription factor (TF) mutant alleles ARepresentative 5‐day‐old seedlings of wild‐type *Arabidopsis thaliana* Col‐0 and TF mutants *gata12* and *wri3* grown in the dark. Scale bar, 1 mm.BHeat map indicates which TFs significantly affect hypocotyl length, root length, and the composite trait of root to total length in dark‐grown seedlings and whether the effects of TFs are dependent on condition. TFs are hierarchically clustered using Euclidean distance.CHeat map of the average relative effects of TF mutant alleles on hypocotyl length, root length, and the ratio of root to total length reveals that TF lesions significantly perturbed TCA cycle‐dependent growth. Mutant alleles are listed in rows and traits in columns. Cells of TF mutant alleles in the heat map are colored if the Arabidopsis Genome Initiative (AGI) or AGI:TCA Metabolite linear model terms for each trait are statistically significant (*P* < 0.05, two‐way ANOVA, 16–20 seedlings per genotype per condition across two experiments per genotype). Mutant alleles are hierarchically clustered using Euclidean distance.D–FHypocotyl length, root length, and the ratio of root to total length are dependent on TF and exogenous TCA cycle metabolites. Radar plots present mutant phenotypes relative to Col‐0 (black). The ratio of mutant: WT (wild‐type) traits was determined using the estimated marginal means (EMMs) of each genotype calculated from 16 to 20 seedlings per genotype per condition across two experiments.

To test the function of TFs within this TCA cycle subnetwork, we developed a system to test the phenotypic consequences of defects in the TCA cycle. Dark‐grown Arabidopsis seedlings utilize the TCA cycle to catabolize seed stores and exogenous carbohydrates and lipids for growth (Padmasree *et al*, 2002; Lee *et al*, 2010; Angelovici *et al*, 2011; Zakhartsev *et al*, 2016). Wild‐type Col‐0 seedlings are etiolated with long hypocotyls and a short root in the dark (Fig 5A). Our previous transcriptome profiling experiment demonstrates that WRI3 is sufficient to regulate TCA cycle gene expression in the dark. In agreement with these data, the hypocotyl and root lengths of the *wri3* loss‐of‐function mutant allele are shorter compared to those of wild type under specific conditions (Fig 5B, C, and E). This suggests that we can utilize hypocotyl and root lengths as a proxy for alterations in the respiratory/TCA cycle output in plant growth and development.

**Figure EV4:**
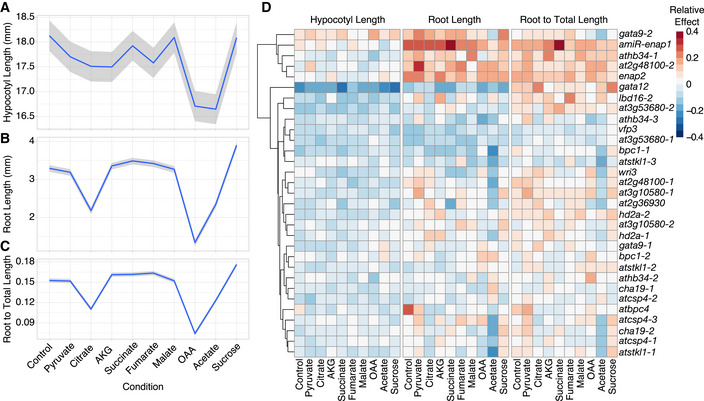
Wild‐type Col‐0 and transcription factor (TF) mutant responses to tricarboxylic acid (TCA) metabolites A–CHypocotyl length, root length, and the ratio of root to total length of *Arabidopsis thaliana* Col‐0 seedlings grown on control or TCA metabolites. Data shown are mean (blue) ± SE (gray) calculated from ˜200 seedlings per condition.DHeat map summarizing the relative effect of TF mutant alleles on the hypocotyl length (left), root length (center), and the ratio of root to total length (right) on control or TCA metabolites‐supplemented media. Mutant alleles are listed in rows.

Transcription factor–TCA cycle target expression correlation analyses suggested that TCA cycle transcriptional regulation is highly context‐dependent. To assess the functional contribution of select TFs from this subnetwork to the TCA cycle and context‐dependent plant growth, we obtained 31 insertional mutant alleles of 17 TFs (Reagents Table). The expression of these 17 TFs was highly correlated with their TCA cycle gene targets in the salt and osmotic stress datasets, and was associated with primary metabolic GOs more than expected by chance (Dataset EV9). To first assess their contribution to the TCA cycle, we measured the hypocotyl and root lengths and the ratio of root length to the total seedling length (the sum of hypocotyl and root lengths) of the mutant alleles. We further queried the dependence of these phenotypes on a particular stage of the TCA cycle by externally supplying TCA cycle intermediates—pyruvate, oxaloacetic acid (OAA), citrate, alpha‐ketoglutarate, succinate, fumarate, and malate. We also included acetate to capture the noncyclic pathway of the TCA cycle and sucrose as a representative respiration‐dependent carbon source (Eastmond *et al*, 2000). In wild‐type seedlings, pyruvate, OAA, citrate, alpha‐ketoglutarate, fumarate, acetate, and sucrose decrease the hypocotyl length; while alpha‐ketoglutarate, succinate, fumarate, malate, and sucrose increase the root length (Fig EV4A and B). The ratio of root to total seedling length of wild‐type *Arabidopsi*s responded similarly to root lengths (Fig EV4C). These observations support our hypotheses that TCA cycle activity and its intermediates have distinct functions in different organs (the root and hypocotyl).

TF‐dependent changes and TF by TCA cycle intermediate‐dependent changes in the three traits were tested using a two‐way ANOVA (Fig 5B, Dataset EV10). Of the 17 tested TFs and their corresponding mutant alleles, 12 TFs have significant genotype‐dependent hypocotyl growth effects. Six TFs have significant genotype‐dependent effects on the root length and seven TFs have significant genotype‐dependent effects on the ratio of root to total seedling length. *HD2A* and *AtHB34* are the two TFs that have significant genotype‐dependent effects across the three measured traits. In the majority of the TF mutant alleles tested, hypocotyl lengths decreased across the TCA intermediate conditions, indicating that the majority of these TFs promote hypocotyl length. The direction of the effect on root length and the ratio of root to total seedling length was more varied among the TF mutant alleles across conditions (Fig EV4D), indicating that the responses to the TCA intermediates in the roots and the ratio of root to total lengths are conditional to the specific TF. Over one third of the TFs have significant effects conditional on TCA intermediate supplementation (Fig 5B). Two TFs showed significant genotype by TCA intermediate effects in hypocotyl length—*GATA12* and *WRI3* (Fig 5B and D). Six of the 17 TFs tested have significant genotype by TCA metabolite effects on root length and root to total length in the mutant allele seedlings, with five TFs common between the two traits (Fig 5B–F, Dataset EV10). In summary, all 17 TFs tested have an effect on plant growth in the dark and TCA‐cycle‐dependent nutrition. Moreover, there was a near‐absence of TFs with significant TCA metabolite interaction effects that overlap between the hypocotyl and the root. This further supports our hypothesis that TCA cycle activity differs dependent on the organ type and points to specific TFs potentially responsible for this conditionality.

### TFs affect salt stress responses

The above phenotypic analyses establish that the TCA cycle TF subnetwork affects TCA cycle‐linked growth and development. We next tested our hypothesis that these TFs also organize TCA cycle function in response to the environment by focusing on salt stress. Salt stress generates a dramatic shift in plant primary metabolism including for the TCA cycle (Sanchez *et al*, 2007). Saline conditions are a major threat to agriculture and we thus analyzed physiologically relevant traits throughout the plant life cycle. All of the 17 TFs are highly correlated with their TCA cycle target genes in salt stress conditions (Dataset EV8). We therefore evaluated the TF mutant alleles’ response to salt stress. At seven days of age, plants were watered with either water or water with 50 mM NaCl. This concentration of salt is the minimum concentration necessary to induce a significant growth difference in wild‐type *Arabidopsis* Col‐0 seedlings (Julkowska *et al*, 2014). Rosette area, growth rate, dry shoot biomass, flowering time, seed yield, and the natural abundance of ^13^C, ^15^N in seeds as well as their ratio (^13^C:^15^N) were measured and provide a comprehensive overview of the agriculturally relevant consequences of TCA cycle disruption over the continuum of plant growth in response to salt stress. TF‐dependent and TF by salt treatment‐dependent responses were evaluated using a two‐way ANOVA (Dataset EV11).

**Figure EV5:**
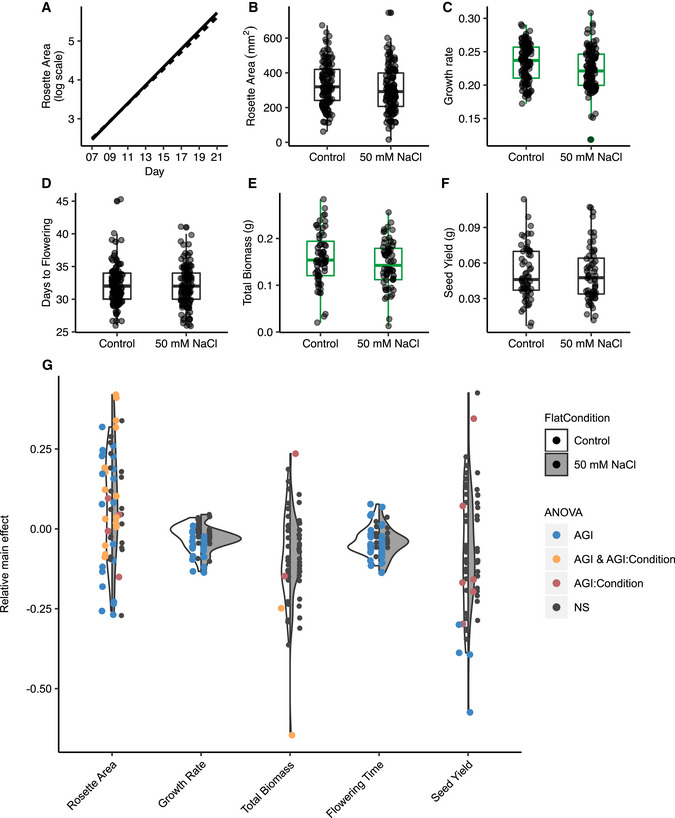
Wild‐type Col‐0 and transcription factor (TF) mutant responses to salt treatment A–FRosette area, growth rate, days to flowering, dry shoot biomass, and seed yield of wild‐type Col‐0 under control and salt conditions. Solid line, control and dashed line, 50 mM NaCl. Green‐colored box plots indicate significant differences between treatments (*P* < 0.05, Student’s *t*‐test, *N* = 100 plants per condition). Box plots mark the interquartile range, from the 25^th^ to the 75^th^ percentile, and are centered at the median. Whiskers extend to 1.5*interquartile range below the lower quartile and above the upper quartile.GDistribution of relative effects of TF mutant alleles under control and salt stress conditions. Dots represent TF mutant alleles. TF mutant alleles are colored if the Arabidopsis Genome Initiative (AGI) (blue), AGI:Condition (red), and AGI and AGI:Condition (orange) linear model terms are significant (*P* < 0.05, two‐way ANOVA). White violin, control condition; gray violin, salt stress condition.

**Figure 6:**
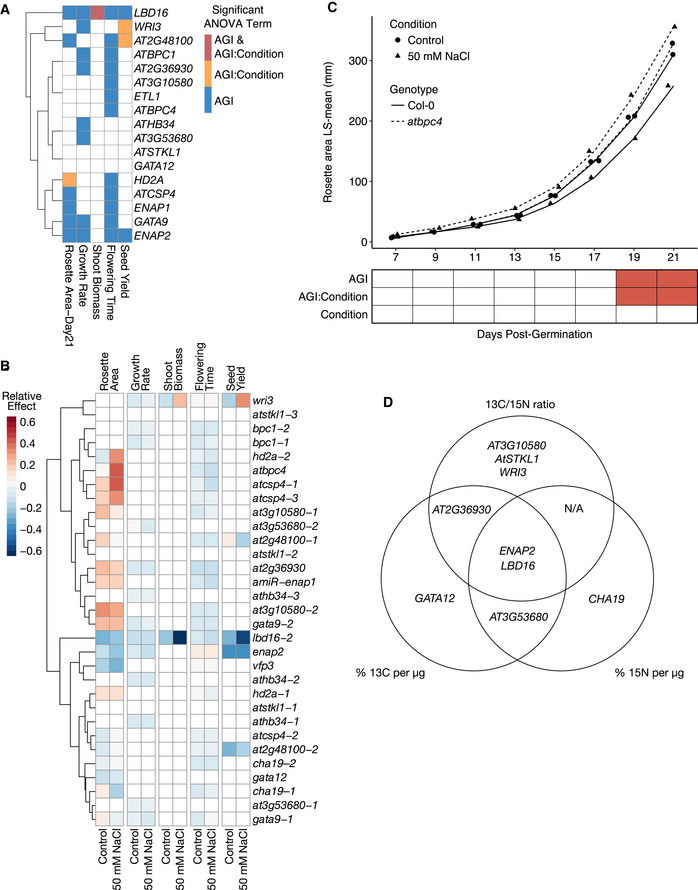
Phenotypes of mutant alleles are genotype by salt treatment‐dependent Heat map indicates which transcription factors (TFs) significantly affect rosette area, growth rate, shoot biomass, flowering time, and seed yield and whether the effects of TFs are dependent on salt treatment. TFs are hierarchically clustered using Euclidean distance.Heat map summarizing the relative effect of TF mutant alleles under control and salt treatment on rosette area, growth rate, shoot biomass, flowering time, and seed yield. Mutant alleles are listed in rows and traits under both control and salt treatment are in columns. Cells of TF mutant alleles in heat map are colored if the Arabidopsis Genome Initiative (AGI) or AGI:Condition term in the linear model of each trait is statistically significant (*P* < 0.05, two‐way ANOVA, *N* = 6–10 plants per genotype per condition). Mutant alleles are hierarchically clustered using Euclidean distance.Rosette area of the mutant allele of *BPC4* from day 7 to day 21 postgermination. Linear model for two‐way ANOVA considers AGI, salt treatment, day postgermination, and their interactions. The AGI and AGI:Salt Condition terms are statistically significant (*P* < 0.001, *P* < 0.01, respectively). Heat map under line plot indicates which term in the linear model is statistically significant (*P* < 0.05) using two‐way ANOVA for each day from nine biological replicates per condition. Solid lines, Col‐0; dashed line, *bpc4*; circle, control condition; triangle, 50 mM NaCl.Venn diagram of transcription factors in which the natural abundance of ^13^C and ^15^N and the ratio of ^13^C to ^15^N that were perturbed in their respective mutant alleles. Transcription factors are listed if the Arabidopsis Genome Initiative (AGI) or AGI:Salt Treatment linear model terms are statistically significant (*P* < 0.05), as determined by a two‐way ANOVA from 3 to 5 biological replicates per genotype per condition.

As anticipated, salt stress significantly reduced growth rate and dry shoot biomass in wild‐type Col‐0 plants (Fig EV5A–F). Mutations in 15 of the TCA cycle‐linked TFs led to small but significant effects on shoot biomass, flowering time, growth rate, and seed yield in both control and salt conditions relative to Col‐0 (Figs 6A and EV5G). These included both positive and negative effects on rosette areas of TF mutants. Six of the TFs tested affected growth in a salt‐dependent manner, including mutants of *BASIC PENTACYSTEINE 4* (*ATBPC4*), *COLD SHOCK DOMAIN PROTEIN 4* (*ATCSP4*), *AT2G48100*, and *CHA19* for rosette area, *LBD16* in dry shoot biomass, and *WRI3* and *AT2G48100* in seed yield (Fig 6B and C, Datasets EV11 and EV12).

**Figure EV6:**
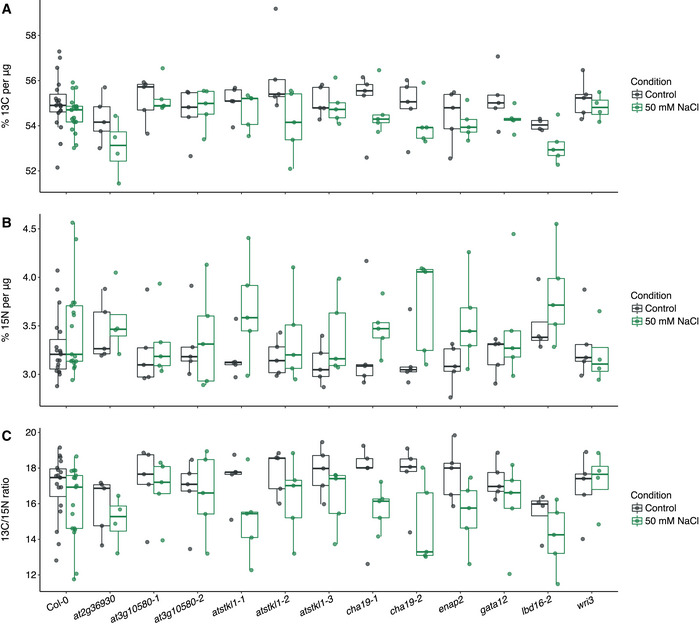
^13^Carbon, ^15^nitrogen and the carbon:nitrogen ratio of transcription factor (TF) mutant alleles A–CPercentages of ^13^C, ^15^N, and the ratio of ^13^C:^15^N of seeds of individuals grown in control and salt stress conditions. Box plots are shaded in if the Arabidopsis Genome Initiative (AGI) or AGI:Condition linear model terms were significant (*P* < 0.05, two‐way ANOVA, *N* = 3–5 biological replicates for the mutant genotype per condition, 17 biological replicates for Col‐0 per condition). Box plots mark the interquartile range, from the 25^th^ to the 75^th^ percentile, and are centered at the median. Whiskers extend to 1.5*interquartile range below the lower quartile and above the upper quartile. Individual measurements are plotted as dots.

In addition to growth phenotypes, mutations in nine of the tested TFs led to significant changes in the abundance of ^13^C, ^15^N, or the ^13^C:^15^N ratio (Fig 6D). Five TFs had significant genotype‐dependent effects on ^13^C abundance. Two TFs had significant genotype‐dependent effects and two TFs had significant TF by salt treatment on ^15^N abundance. Among the TFs that affected the ^13^C:^15^N ratio, two TFs had significant genotype‐dependent effects and three TFs had significant effects dependent on the salt treatment (Fig 6D, Datasets EV11 and EV12). Mutations in *ENAP2* and *LBD16* generated significant differences in all three isotope abundance traits relative to Col‐0 (Figs 6D and EV6, Datasets EV11 and EV12).

**Figure EV7:**
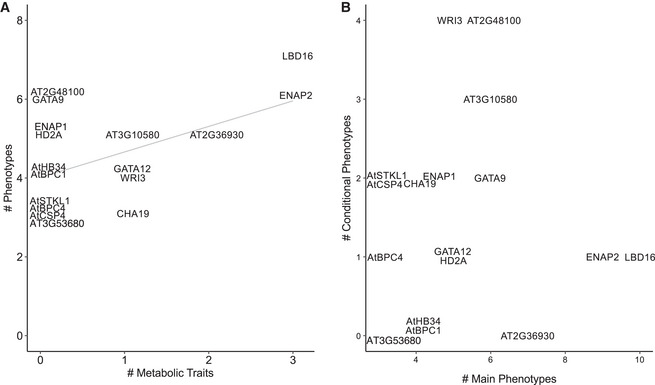
Summary of transcription factor (TF) mutant growth traits and metabolic phenotypes A positive correlation is observed between the number of significant growth phenotypes from the tricarboxylic acid (TCA) metabolite feeding and salt stress experiments and the number of significant metabolic phenotypes of seeds from the salt stress experiment (*r* = 0.53, *R* ^2^ = 0.28, *P* = 0.03, Pearson correlation).No correlation is observed between the total number of TFs with significant main effects and the total number of TFs with significant conditional (interaction) effects (*r* = 0.034, *R* ^2^ = 0.0011, *P* = 0.90, Pearson correlation).

Given the influence of these TFs on both dark‐mediated growth and salt stress, we investigated if the two sets of traits showed a connection as might be expected if a single metabolic pathway (the TCA cycle) underlies both context‐dependent phenotypes. A positive correlation (*r* = 0.53, *P* = 0.03) was observed between the number of significant phenotypes observed in the dark TCA intermediate‐feeding and salt stress response experiments and the number of significant C and N content phenotypes (Fig EV7). In summary, our phenotypic analyses of mutant alleles of 17 TFs within this TCA cycle subnetwork demonstrate this regulatory module’s importance in plant growth and its response to salt stress as well as the importance of TFs in coordination of TCA cycle function in different organs and conditions.

## Discussion

Here, we present a global map of interactions between TFs and gene promoters in central carbon and specialized metabolism in *Arabidopsis*. The Y1H methods are complementary to other high‐throughput *in vitro* assays including protein‐binding microarrays (Weirauch *et al*, 2014), DAP‐seq (O'Malley *et al*, 2016), or computational inferences (Kulkarni *et al*, 2018) to identify interactions between TFs and target genes. Some of these methods assay for interactions between hundreds of TFs—916 TFs (Kulkarni *et al*, 2018) or 529 TFs (O'Malley *et al*, 2016). This mapped set of interactions utilizes a full TF collection, which has been utilized in Y1H assays (Breton *et al*, 2016; Bonaldi *et al*, 2017; Kang *et al*, 2018; Li *et al*, 2019), albeit with fragments of single regulatory regions. Here, we utilized an enhanced Y1H system (Gaudinier *et al*, 2011) to screen for interactions with 224 promoters enabling the detection of a vastly higher proportion of transcriptional regulators of primary and specialized metabolism than these previously mentioned studies.

The resulting putative regulatory network model differs from that found in single‐celled organisms. In this network, most TFs bind to promoters of genes in two or more metabolic pathways rather than being pathway‐specific TFs, suggesting a model of regulation that requires TF coordination of multiple pathways. Additionally, a large collection of TFs were identified via the Y1H analyses that could potentially regulate each pathway. Together, these analyses suggest that a pathway‐specific or master‐regulatory model is not likely the design principle underlying metabolism in *Arabidopsis*, and potentially, plants. Instead, we propose that plant metabolism is controlled via a distributed system whereby each pathway is influenced by a large collection of TFs. This collection of TFs provides the ability for multiple pathways to be coordinated as a unit and for different modules to be created. This potential was supported by the inducible TF analyses whereby genes in the pentose phosphate pathway, glycine biosynthesis, and cysteine biosynthesis were differentially expressed upon induction of each single TF, with enrichment for their putative regulatory targets.

An additional benefit of a distributed control system is that it allows for more fine‐tuning to precisely respond to either environmental or developmental cues. This potential has been previously observed in the aliphatic GSL pathway wherein TFs had tissue‐ and/or environmentally conditional effects (Li *et al*, 2014, 2018). In this study, we were able to extend this observation of developmental and environmental context‐specific regulation of the TCA cycle, a central primary metabolic pathway within *Arabidopsis*. Genetic perturbation of 17 TFs revealed that these factors regulate the TCA cycle and the localization of their targets suggests differential transcriptional control of TCA cycle targets within various cellular compartments, potentially increasing the flexibility of the pathway. These findings on the TCA cycle further enhance the difference in multicellular metabolic regulation as the TCA cycle within the single‐celled *Escherichia coli* and *Saccharomyces cerevisiae* is determined by a limited number of metabolites using a few key TFs.

This resource of TF–enzyme promoter interactions in *Arabidopsis* has allowed us to propose a hypothesis that multicellular organisms rely on a distributed regulatory system for controlling metabolism rather than the previously observed master‐regulatory/pathway‐specific system found in single‐celled organisms. Furthermore, it can provide an important source of regulatory modules to enable combinatorial engineering of plant metabolism across diverse metabolic pathways.

## Materials and Methods

### Reagents and Tools table

**Table:** 

Reagent/Resource	Reference or Source	Identifier or Catalog number
**Experimental Models**		
*Arabidopsis thaliana* Col‐0	Dr. Daniel Kliebenstein (UC Davis)	N/A
*Arabidopsis thaliana wri3,* AT1G16060	*Arabidopsis* Biological Resource Center	SALK_144578
*Arabidopsis thaliana bpc1‐1,* AT2G01930	*Arabidopsis* Biological Resource Center	SALK_072966
*Arabidopsis thaliana bpc1‐2,* AT2G01930	*Arabidopsis* Biological Resource Center	SALK_101466
*Arabidopsis thaliana cha19‐2,* AT2G02090	*Arabidopsis* Biological Resource Center	SALK_069014
*Arabidopsis thaliana cha19‐1,* AT2G02090	*Arabidopsis* Biological Resource Center	SALK_054130
*Arabidopsis thaliana atcsp4‐1,* AT2G21060	*Arabidopsis* Biological Resource Center	GABI‐579G10
*Arabidopsis thaliana atcsp4‐2,* AT2G21060	*Arabidopsis* Biological Resource Center	GABI_623B08
*Arabidopsis thaliana atcsp4‐3,* AT2G21060	*Arabidopsis* Biological Resource Center	SAIL_858_A06
*Arabidopsis thaliana atbpc4,* AT2G21240	*Arabidopsis* Biological Resource Center	SALK_014313
*Arabidopsis thaliana at2g36930*	*Arabidopsis* Biological Resource Center	SALK_209159C
*Arabidopsis thaliana lbd16‐2,* AT2G42430	*Arabidopsis* Biological Resource Center	SALK_040739C
*Arabidopsis thaliana at2g48100‐1*	*Arabidopsis* Biological Resource Center	SALK_009105
*Arabidopsis thaliana at2g48100‐2*	*Arabidopsis* Biological Resource Center	GABI_190A05
*Arabidopsis thaliana at3g10580‐1*	Arabidopsis Biological Resource Center	SALK_000108
*Arabidopsis thaliana at3g10580‐2*	Arabidopsis Biological Resource Center	SALK_020321
*Arabidopsis thaliana amiR‐enap1,* AT3G11100	Zhang *et al* (2016); provided by Dr. Hong Qiao (UT Austin)	N/A
*Arabidopsis thaliana enap1,* AT3G11100	Arabidopsis Biological Resource Center	GABI_423B09
*Arabidopsis thaliana athb34‐1,* AT3G28920	Arabidopsis Biological Resource Center	SALK_085482C
*Arabidopsis thaliana athb34‐2,* AT3G28920	Arabidopsis Biological Resource Center	SALK_123593C
*Arabidopsis thaliana athb34‐3,* AT3G28920	Arabidopsis Biological Resource Center	SALK_147851C
*Arabidopsis thaliana hd2a‐1,* AT3G44750	Arabidopsis Biological Resource Center	GABI_355H03
*Arabidopsis thaliana hd2a‐2,* AT3G44750	Arabidopsis Biological Resource Center	SAIL_906_B06
*Arabidopsis thaliana at3g53680‐1*	Arabidopsis Biological Resource Center	SALK_117411
*Arabidopsis thaliana at3g53680‐2*	Arabidopsis Biological Resource Center	GABI_180C10
*Arabidopsis thaliana atstkl1‐1,* AT4G00238	Arabidopsis Biological Resource Center	SALK_083259
*Arabidopsis thaliana atstkl1‐2,* AT4G00238	Arabidopsis Biological Resource Center	SALK_019920
*Arabidopsis thaliana atstkl1‐3,* AT4G00238	Arabidopsis Biological Resource Center	SALK_068662
*Arabidopsis thaliana gata9‐2,* AT4G32890	Arabidopsis Biological Resource Center	SALK_080142C
*Arabidopsis thaliana gata9‐1,* AT4G32890	Arabidopsis Biological Resource Center	SALK_152156
*Arabidopsis thaliana enap2,* AT5G05550	Zhang *et al* (2016); provided by Dr. Hong Qiao (UT Austin)	N/A
*Arabidopsis thaliana gata12,* AT5G25830	Arabidopsis Biological Resource Center	SALK_052546
*Arabidopsis thaliana p35S::GR‐CHA19* line 5	This paper	N/A
*Arabidopsis thaliana p35S::GR‐CHA19* line 7	This paper	N/A
*Arabidopsis thaliana p35S::GR‐CHA19* line 8	This paper	N/A
*Arabidopsis thaliana p35S::GR‐CHA19* line 9	This paper	N/A
*Arabidopsis thaliana p35S::GR‐ENAP1* line 1	This paper	N/A
*Arabidopsis thaliana p35S::GR‐ENAP1* line 2	This paper	N/A
*Arabidopsis thaliana p35S::GR‐ENAP1* line 4	This paper	N/A
*Arabidopsis thaliana p35S::GR‐ENAP1* line 5	This paper	N/A
*Arabidopsis thaliana p35S::GR‐LBD16* line 3	This paper	N/A
*Arabidopsis thaliana p35S::GR‐LBD16* line 8	This paper	N/A
*Arabidopsis thaliana p35S::GR‐LBD16* line 11	This paper	N/A
*Arabidopsis thaliana p35S::GR‐LBD16* line 12	This paper	N/A
*Arabidopsis thaliana p35S::GR‐WRI3* line 8	This paper	N/A
*Arabidopsis thaliana p35S::GR‐WRI3* line 10	This paper	N/A
*Arabidopsis thaliana p35S::GR‐WRI3* line 17	This paper	N/A
*Arabidopsis thaliana p35S::GR‐WRI3* line 21	This paper	N/A
**Recombinant DNA**		
TOPO‐U Arabidopsis TF ORFeome	Arabidopsis Biological Resource Center; Pruneda‐Paz *et al* (2014); see Table EV2	CD4‐88
pMW#2	Deplancke *et al* (2006); provided by Dr. Marian Walhout (U Mass Medical)	N/A
pMW#3	Deplancke *et al* (2006); provided by Dr. Marian Walhout (U Mass Medical)	N/A
pDest‐AD‐2μ	Reece‐Hoyes *et al* (2011a); provided by Dr. Marian Walhout (U Mass Medical)	N/A
pFAST_R05	Shimada *et al* (2010); Gateway Vectors	3_75
pBeaconRFP_GR	Bargmann *et al* (2013); Gateway Vectors	5_68
**Oligonucleotides and sequence‐based reagents**		
Promoter cloning primers	This study	Table EV1
T‐DNA insertional mutant genotyping primers	This study	Table EV13
LBb1.3: ATTTTGCCGATTTCGGAAC	IDT	N/A
**Software**		
emmeans v1.4	Length (2016), CRAN	
FastQC v.0.11.7	https://www.bioinformatics.babraham.ac.uk/projects/fastqc/	
TrimGalore v0.6.0	http://www.bioinformatics.babraham.ac.uk/projects/trim_galore/	
STAR aligner v2.7.0f	Dobin *et al* (2013)	
octopus v0.3.7	Zhang *et al* (2017), https://github.com/WeiZhang317/octopus	
Cytoscape v3.7.1	Shannon *et al* (2003), www.cytoscape.org	
Affy	Bioconductor v3.4, http://bioconductor.org/packages/release/bioc/html/affy.html	
**Other**		
Developmental Atlas dataset	Schmid *et al*, Array Express E‐TABM‐17	https://www.ebi.ac.uk/arrayexpress/experiments/E‐TABM‐17/
Root developmental atlas dataset	Brady *et al* (2007), Birnbaum *et al* (2003), Lee *et al* (2006), Levesque *et al* (2006), AREX	http://www.arexdb.org/download.html
Pollen developmental atlas dataset	Honys and Twell (2004), Gene Expression Omnibus	GSE6162
Pollen developmental atlas dataset	Qin *et al* (2009), Gene Expression Omnibus	GSE17343
Salt stress microarray dataset	Kilian *et al* (2007), Gene Expression Omnibus	GSE5623
Osmotic stress microarray dataset	Kilian *et al* (2007), Gene Expression Omnibus	GSE5622
GR‐TF transcriptome	This paper; Gene Expression Omnibus	GSE137623

### Methods and Protocols

#### *Saccharomyces cerevisiae* (Transformation)

*Saccharomyces cerevisiae* strain YM4271 (for promoters) or strain Yα1867 (for TFs) was grown overnight at 30°C by taking streaking from a glycerol stock onto a YPDA (yeast peptone dextrose adenine) agar plate to make a lawn. A pea‐sized glob of yeast cells was resuspended in 1 ml of liquid YPDA and ˜100 μl was added to 50 ml of liquid YPDA in an Erlenmeyer flask to a starting OD_600_ of 0.15–0.20. The yeast culture was grown in a 30°C shaking incubator at 210 rpm for 2 h or until OD_600_ had reached 0.4–0.6.

#### *Saccharomyces cerevisiae* (Y1H)

Five ml of SC–HIS–URA was inoculated with yeast strains of the promoters from glycerol stock and grown at 30°C and shaking at 210 rpm for 48 h. Cultures of promoter yeast strains were concentrated by pelleting down yeast cells (centrifuge for 5 min at 1,850 *g*) and resuspending in 1–1.5 ml of liquid SC–HIS–URA, and then 510 μl spread onto SC–HIS–URA agar plates with 15–20 glass beads. Promoter lawn plates were incubated for two nights at 30°C. The TF collection was cultured in deep 96‐well plates from glycerol stocks in 315 μl of liquid SC‐TRP for two nights at 30°C. TF culture plates were spun down for 5 min at 1,850 *g* and 150 μl of liquid media was removed. The pellets were resuspended in the remaining liquid media and 50 μl of the culture transferred to 384‐well plate and arrayed in duplicates, resulting in two 96‐well plates combined into one 394‐well plate.

#### *Arabidopsis thaliana* (Bulking and genotyping)

Transfer DNA (T‐DNA) insertional mutant lines were ordered from the Arabidopsis Biological Resource Center at Ohio State University (Alonso *et al*, 2003) or obtained from published sources (Zhang *et al*, 2016). T‐DNA insertional mutant lines were confirmed by polymerase chain reaction (PCR) using LP + RP + LBb1.3 primers (Dataset EV13). To minimize variation due to maternal conditions, all mutant lines were bulked together with wild‐type except for *at2g48100‐2*, *enap1*, and *gata12* (Reagents Table), which were bulked together with another growth of the wild‐type reference. *Arabidopsis* plants were grown in Sunshine mix #1 in 2.23” × 1.94” × 2.23” pots. The chamber temperature was set to continuous 22°C and set to long day (16‐h light/8‐h dark) with light intensity of ˜120 μmol m^−2^ s^−1^ from T12 very high output (VHO) fluorescent light bulbs. Seeds were sieved for sizes between 250 and 300 μm and surface sterilized by soaking in 50% household bleach, 0.05% Tween 20 for 20 min and then rinsed with sterile water 5–7 times before stratifying in 0.1% sterile agar for 2 days in 4°C. Seeds were sown in Sunshine Mix #1 in traditional 1,020 trays with 18‐cell inserts (pot dimension 3.10” × 3.10” × 2.33”).

#### *Arabidopsis thaliana* (TCA metabolite feeding)

Seeds were plated onto half‐strength Murashige and Skoog (MS) agar pH 5.7 with potassium hydroxide (KOH), supplemented with 1 mM TCA metabolites or 0.21 mM sucrose based on theoretical ATP yield (Rich, 2003). The concentration of sucrose was calculated to provide an equivalent theoretical yield of ATP as 1 mM of the TCA metabolites. The pH of 0.25 M OAA, ketoglutaric acid, and pyruvic acid were adjusted with KOH, filter sterilized (0.22 μm), and then added to MS agar after autoclaving to a final concentration of 1 mM. The plates were then wrapped in double layers of aluminum foil and grown standing vertically for 5 days in the dark in ambient room temperature (21–23°C). Germination was determined by unwrapping additional plates each day postplating.

#### *Arabidopsis thaliana* (Salt response)

Seeds were sieved for sizes between 250 and 300 μm and surface sterilized by soaking in 50% household bleach, 0.05% Tween 20 for 20 min and then rinsed with sterile water 5–7 times before stratifying in 0.1% sterile agar for 2 days in 4°C. The seeds were sown in Sunshine Mix #1 in traditional 1,020 trays with 18‐cell inserts (pot dimension 3.10” × 3.10” × 2.33”) and grown in a chamber with the temperature set to continuous 22°C and set to long day (16‐h light/8‐h dark) with light intensity of ˜120 μmol m^−2^ s^−1^ from T12 VHO fluorescent light bulbs. After germination, each pot was thinned to one seedling per pot. When the seedlings were 7 days old, flats were watered every 5–7 days with either deionized water (control) or 50 mM NaCl (liquid; treatment) to maintain soil moisture.

#### *Arabidopsis thaliana* (Transformation)

Ten to twelve *Arabidopsis thaliana* Col‐0 seeds were sown in Sunshine Mix #1 in 3.5” × 3.5” × 5” pots. Pots were incubated at 4°C for 3 days before transferring into a growth chamber set to continuous 22°C and set to long day (16‐h light/8‐h dark) with light intensity of ˜120 μmol m^−2^ s^−1^ from T12 VHO fluorescent light bulb. Five days after seed germination, each pot was thinned to five seedlings per pot and grown to four to five weeks for *Agrobacterium* transformation.

#### *Arabidopsis thaliana* (RNA‐Seq)

T3 seeds from four independent lines were selected by the presence of red fluorescent protein (RFP) in the seed. Approximately 200–300 seeds of each line were surface sterilized with 50% bleach for 15 min, rinsed 5–7 times with sterile distilled water, and imbibed in the dark at 4°C for two days. The seeds were then sowed onto nylon mesh on Petri plates containing half‐strength MS agar plates pH 5.7 with KOH. The plates were double wrapped in foil and placed vertically in a dark chamber at ambient room temperature (20–22°C).

#### Network visualization

All networks are visualized using Cytoscape v3.7.1 (Shannon *et al*, 2003).

#### Bait promoter cloning

The PCR primers were designed to amplify promoter regions of 2,000 bp in size or to the next gene upstream from the predicted translational start site of each gene (Dataset EV1). Promoter regions were amplified from Col‐0 genomic DNA or from plasmids containing 1 kb synthesized promoters (Life Technologies) using Phusion High‐Fidelity Taq (NEB) and cloned into pENTR 5’ TOPO vector (Invitrogen). Promoter regions were then recombined with pMW2 and pMW3 destination vectors (Deplancke *et al*, 2006) in LR reactions and sequence‐confirmed before transforming into the YM4271 yeast strain as described in (Gaudinier *et al*, 2011).

#### Prey TF cloning

Complementary DNA (cDNA) of TFs in pENTR from (Pruneda‐Paz *et al*, 2014) that were not present in the root‐expressed TF collection (Gaudinier *et al*, 2011) were recombined with pDEST‐AD‐2 micron destination vector in LR reactions. Prey TFs were transformed into Yα1867 yeast strain.

#### Yeast one‐hybrid

All promoter baits were screened against a total collection of 2039 prey TFs, according to protocols in (Gaudinier *et al*, 2011, 2017).

#### Pairwise pathway comparison

To determine the significance of association between two metabolic pathways based of their TF binding, we performed a one‐tailed Fisher’s exact test using fisher.test in R version 3.4.0. A 2 × 2 contingency matrix is calculated for all pairwise combinations of metabolic pathways based on the number of TFs detected in both pathways, the number of TFs detected in the first pathway, the number of TFs detected in the second pathway, and the number of TFs screened (2039 TFs). *P*‐values were adjusted for multiple testing using p.adjust in R version 3.4.0. Regional enrichment for aliphatic GSL biosynthesis was defined by the rank order of (adjusted p‐values/odds ratios).

#### TF family enrichment

Subcellular localization of TCA cycle targets was determined from the SUBA3 database (Tanz *et al*, 2013). The consensus‐predicted subcellular localization was used, except when a gene’s subcellular localization was previously experimentally tested as per the literature. In that case, the experimentally determined subcellular localization was used. The number of interactions was obtained for each TF family targeting a TCA cycle enzymatic step in each cellular compartment from the Y1H interaction data. TF family enrichment was tested using a two‐tailed Fisher’s exact test with Holm–Bonferroni to adjust for multiple testing in R. The number of TFs in each cellular compartment in each family was tested against the number of TFs in the Y1H assay.

#### Correlation analysis

The Y1H data generated a network of TF–TCA cycle target gene interactions, where TF or target genes were nodes and interactions were edges. Using this network structure, Pearson correlation coefficients were calculated for each TF–TCA cycle gene interaction in R using the cor function with gene expression values from publicly available microarray datasets (Reagents Table). These datasets capture a range of experimental and biological perturbations and thus allow for the capture of conditional transcriptional correlations. We downloaded available.CEL files from the experiments only when the genotype corresponded to *Arabidopsis thaliana* accession Col‐0. This was to exclude the complications of polygenic natural variation between different accessions and to focus on perturbations in a single genotype. Using the.CEL files, the microarray datasets were normalized using the rma function from the affy package (Gautier *et al*, 2004) in Bioconductor.

We parsed these datasets to represent specific aspects of developmental biology or stress response. The first was the *Arabidopsis* development (Schmid *et al*, 2005) and root development expression atlases (Birnbaum *et al*, 2003; Lee *et al*, 2006; Levesque *et al*, 2006; Brady *et al*, 2007) that encompass numerous cell‐, tissue‐, and organ‐types. We added the pollen development expression atlas (Honys & Twell, 2004; Qin *et al*, 2009) because of the coordinated transcriptional changes with strong temporal regulation observed in pollen metabolism, across pollen maturation and pollen germination. In addition to development, we included a large salt and osmotic stress (Kilian *et al*, 2007) expression atlas because these stresses modulate photosynthesis and cellular respiration associated with the TCA cycle.

#### Gene ontology enrichment analysis

Arabidopsis Genome Initiative (AGI) loci identifiers were uploaded to the GO Term Enrichment tool powered by PANTHER released July 11, 2019 on www.arabidopsis.org. The GO term enrichment analysis was based on GO biological process complete annotations from the Gene Ontology database released October 08, 2019.

#### TCA metabolite feeding assay

For each experiment, 30 replicates of Col‐0 and 10 mutants were plated in a random block design. The TF mutant lines were divided into two blocks. Each plate (block) had six wild‐type seedlings and one seedling per mutant line for 15 or 16 TF mutant genotypes plated across three rows. The entire experiment was repeated twice, for a total of 20 biological replicates maximum per TF mutant lines and 60 biological replicates maximum of Col‐0 per TCA metabolite. Backlit images of plates on a light table were acquired using a Canon EOS T3i Rebel dSLR camera fixed on a camera stand. Roots and hypocotyls were traced manually using a Wacom Intuos drawing tablet in ImageJ.

#### Salt response assay

Mutant seedlings were grown in a random block design. Each block consists of 10–11 mutant TF lines plus Col‐0, for a total of three blocks. Each experiment consists of five biological replicates per TF mutant lines and the entire experiment was repeated twice for a total of 10 biological replicates maximum per TF mutant. The concentration of salt selected was the minimum concentration necessary to induce a significant growth difference in wild‐type *Arabidopsis* Col‐0 seedlings (Julkowska *et al*, 2014). A copy stand and Canon T3 dSLR were used to take aerial images of each flat every other day starting at 7 days postgermination until 21 days postgermination. Each image had a measurement marker to standardize size images between pictures. Image analysis to obtain rosette area was conducted in ImageJ using the Analyze Particle function after selecting Hue (42–166), Saturation (28–255), and Brightness (80–255) under Adjust > Color Threshold. The dry shoot biomass was determined from fully mature shoots from 3 to 5 individuals of each genotype at the end of the first salt stress experiment. The shoots were dried in a 60°C oven overnight and then weighed on an analytical balance.

#### Natural ^13^C and ^15^N abundance profiling

For Carbon‐13 and Nitrogen‐15 profiling, mature seeds from 3 to 5 individuals of each genotype were sieved and cleaned to remove any plant material and then dried in a 60°C oven overnight. The seeds were obtained from the control and salt conditions from the first salt stress experiment. The seeds were allowed to acclimate to ambient room conditions before 2–3 mg of seeds was submitted to the Stable Isotope Facility at the University of California, Davis (UC Davis) for determining the natural abundance of Carbon‐13 and Nitrogen‐15.

#### Statistical analysis for T‐DNA insertional mutant phenotype assays

Replication numbers for all experiments were designed to provide significant power for moderate effect sizes and modest power for small effect sizes using a presumed broad‐sense heritability of about 15% based on previous experience with these traits. All statistical analyses were conducted in R version 3.4.0. The TFs controlling the root length, hypocotyl lengths, root to total seedling lengths, rosette area, vegetative growth rate, dry shoot biomass, flowering time, and seed yield were tested by ANOVA using a general linear regression model in R with the package lmerTest. The following generalized nested linear mixed model was used: Trait = AGI + AGI:Allele + Treatment + AGI:Treatment + AGI:Allele:Treatment. In the case of rosette area, day was included as a factor: Trait = AGI + AGI:Allele + Treatment + Day + AGI:Day + AGI:Treatment + Treatment:Day + AGI:Allele:Treatment + AGI:Treatment:Day. The random‐effect terms for the dark feeding experiment are Block nested in Experiment and Row on Plate nested in Plate. The random‐effect terms for the salt response experiment are Flat Condition nested in Shelf nested in Experiment. Random‐effect terms in the models were computed using ranova function from the lmerTest package. To allow for an AGI:Allele term in the generalized linear model, growth traits (hypocotyl lengths, root lengths, and rosette areas) of the two alleles that were bulked at a different time were normalized by adding the difference between the two wild types to the raw values. Estimated marginal means (EMMs/least‐squares means) and *post hoc* comparisons between mutant alleles and Col‐0 conditioned on treatment were calculated using the emmeans (Lenth, 2016) package in R. P‐values from *post hoc* tests were adjusted for multiple comparisons using the Holm method in R. The relative effect is expressed as EMM_Mutant_ – EMM_WT_/EMM_WT_.

#### Cloning dexamethasone‐inducible overexpression TF lines

The coding sequences of *CHA19*, *ENAP1*, *LBD16*, and *WRI3* in pENTR from the *Arabdiopsis* TF ORFeome collection (Pruneda‐Paz *et al*, 2014) (CD4‐88) were recombined into a modified pFAST‐R05 plasmid with the dexamethasone‐inducible GR construct from pBeaconRFP‐GR plasmid using LR reactions. To generate the modified vector, pFAST‐R05 (Shimada *et al*, 2010) was digested with SbfI and ApaI to serve as the backbone of the modified plasmid, containing the LB, RB, and pOLE1:OLE1‐RFP, a seed‐selectable RFP marker. The dexamethasone‐inducible overexpression cassette from pBEACONRFP–GR (Bargmann *et al*, 2013) was cloned using Phusion High Fidelity Taq (Forward primer: GACTAGAGCCAAGCTGATCTCC; Reverse primer: CGACGTCGCATGCCTGCAGG), sequenced verified, and then recombined into the digested pFAST‐R05 backbone using the Gibson assembly (NEB E2611S) (Forward primer: CCTGCAGGCATGCGACGTCGTCAAGCTTAGCTTGAGCTTGGATCA; Reverse primer: CAAGCTCAAGCTAAGCTTGACGACGTCGCATGCCTGCAGG). Recombined TFs in the modified pFAST‐R05 with the dexamethasone‐inducible GR system were sequence‐confirmed and then transformed into *Agrobacterium tumefaciens* strain EHA105 using the calcium chloride freeze‐thaw method (Holster *et al*, 1978). Transformed *Agrobacterium* were selected on Luria‐Bertani (LB) agar plates containing spectinomycin and rifampicin and then confirmed by genotyping polymerase chain. Four‐ to five‐week‐old, flowering *Arabidopsis thaliana* Col‐0 were transformed using the floral dip method (Clough & Bent, 1998). The transformants were selected by the presence of RFP in the seed coat using a 532‐nm green laser and red filter, and confirmed by genotyping PCR. The transformants were bulked together to T3 under the same conditions as the T‐DNA insertional mutant lines.

#### GR induction and RNA‐Seq library construction

At day 5, the seedlings on the nylon mesh were transferred onto mock plates or half‐strength MS agar plates containing 10 μM dexamethasone (Dex) in the dark, assisted with a green headlamp. After 24 h of induction, the whole seedlings were collected, flash frozen in liquid nitrogen, and stored in −80°C until RNA‐Seq library constructions. Direct mRNA isolation was performed using biotinylated polyT oligonucleotide and streptavidin‐coated magnetic beads following the protocol from (Townsley *et al*, 2015). Nonstrand‐specific RNA‐Seq libraries were prepared, according to the protocol for the high‐throughput RNA‐Seq library preparation method (Kumar *et al*, 2012). A total of 64 RNA‐Seq libraries were made, representing two biological replicates for the two treatments across four independent Dex‐controlled GR–TF mutant lines (Reagents Table). Libraries were sequenced twice on the Illumina HiSeq 4000 system in the SR100 mode at the University of California, Davis DNA Technologies Core.

#### RNA‐Seq analysis

FastQ file processing was performed on the University of California, Davis high performance bioinformatics cluster. FastQ files were quality checked using FastQC v.0.11.7 (https://www.bioinformatics.babraham.ac.uk/projects/fastqc/), adapter and poly‐A trimmed using TrimGalore v0.6.0 using default settings (http://www.bioinformatics.babraham.ac.uk/projects/trim_galore/) and then reassessed for quality using FastQC. Gene counts were obtained by mapping trimmed reads to the *Arabidopsis thaliana* genome (TAIR10) using STAR aligner v2.7.0f (Dobin *et al*, 2013). Assessing that the read count and quality were similar across the two sequencing runs, gene counts were summed up. Statistical analysis was performed in R 3.6.0 using the octopus pipeline established in (Zhang *et al*, 2017) (https://github.com/WeiZhang317/octopus). Briefly, trimmed mean of M value normalization was performed using the calcNormFactors function from edgeR (Robinson *et al*, 2010) version 3.26.6. For each TF, we ran the following negative binomial generalized linear model using the glm.nb function from MASS package to test for genes that were significantly influenced by the translocation of the TF into the nucleus: *Y = T_I_ + A_L_ + T_I_ × A_L_* where the main effects T and A are denoted as dexamethasone treatment and allele of TF, respectively. The false discovery rate was corrected using Benjamini–Hochberg with the p.adjust function. GO analysis was conducted on pantherdb.org by submitting the list of AGIs of genes significantly differentially expressed due to translocation of the GR–TFs’ chimeric protein into the nucleus. The complete biological process GO annotation dataset for *Arabidopsis thaliana* was used for testing statistical overrepresentation. Enrichment analysis was performed in R using the fisher.test function.

## Author contributions

DJK, SMB, MT, and BL conceived and designed the study. DJK and SMB secured funding for the project. MT, BL, XZ, TB, JJL, NC, AG, RN, and CC‐W performed the experiments and generated the data. MT conducted the formal analysis. MT, SMB, and DJK wrote the manuscript with contributions from all authors. DJK and SMB supervised the project.

## Conflict of interest

The authors declare that they have no conflict of interest.

## Supporting information



Expanded View Figures PDF

Dataset EV1

Dataset EV2

Dataset EV3

Dataset EV4

Dataset EV5

Dataset EV6

Dataset EV7

Dataset EV8

Dataset EV9

Dataset EV10

Dataset EV11

Dataset EV12

Dataset EV13

## Data Availability

The datasets and computer code produced in this study are available in the following databases:
RNA‐Seq data: Gene Expression Omnibus GSE137623 (https://www.ncbi.nlm.nih.gov/geo/query/acc.cgi?acc=GSE137623). Raw data files and analysis scripts: GitHub (https://github.com/melletang/ccp_y1h).
