# Supplementary material for: A genome‐scale TF–DNA interaction network of transcriptional regulation of Arabidopsis primary and specialized metabolism
Source: Mol Syst Biol. 2021 Nov 24;17(11):e10625. doi: 10.15252/msb.202110625 (PMC8611409; doi:10.15252/msb.202110625)
Supplement: Supplementary file 1 — Expanded View Figures PDF [file MSB-17-e10625-s008.pdf]

Expanded View Figures

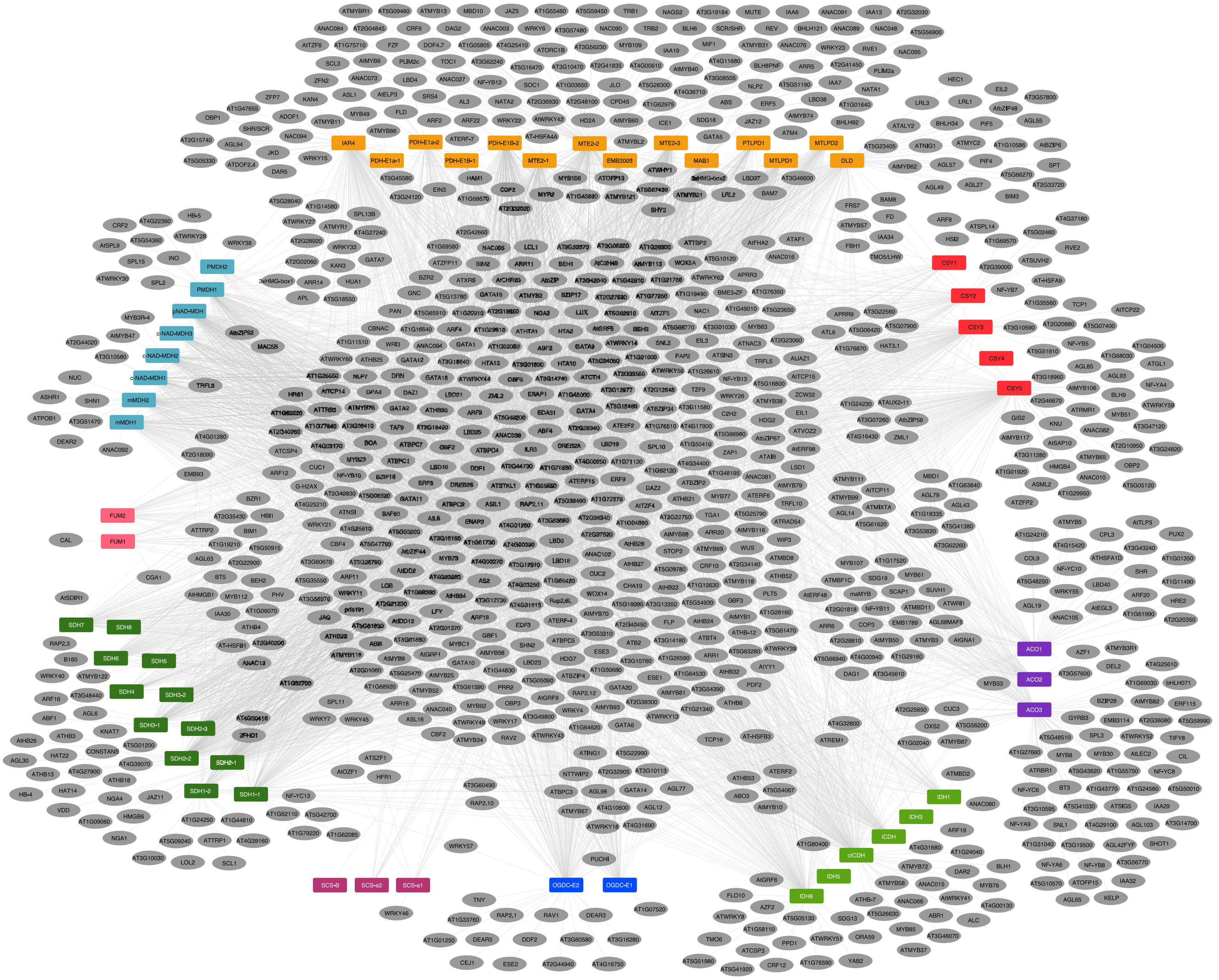

Figure EV1. Transcription factor–tricarboxylic acid (TF–TCA) cycle target gene interaction network.

Yeast one-hybrid (Y1H) assays revealed a large and combinatorial network of TF–TCA cycle target gene interactions. Colored rectangles, promoters; gray oval, transcription factor; gray edge, interaction. Orange PDC, pyruvate dehydrogenase; red CSY, citrate synthase; purple ACO, aconitase; light green IDH, isocitrate dehydrogenase; blue OGD, oxoglutarate dehydrogenase; light purple SCL, succinyl CoA ligase; green SDH, succinate dehydrogenase; pink FUM, fumarase; light blue MDH, malate dehydrogenase.

 © 2021 The Authors

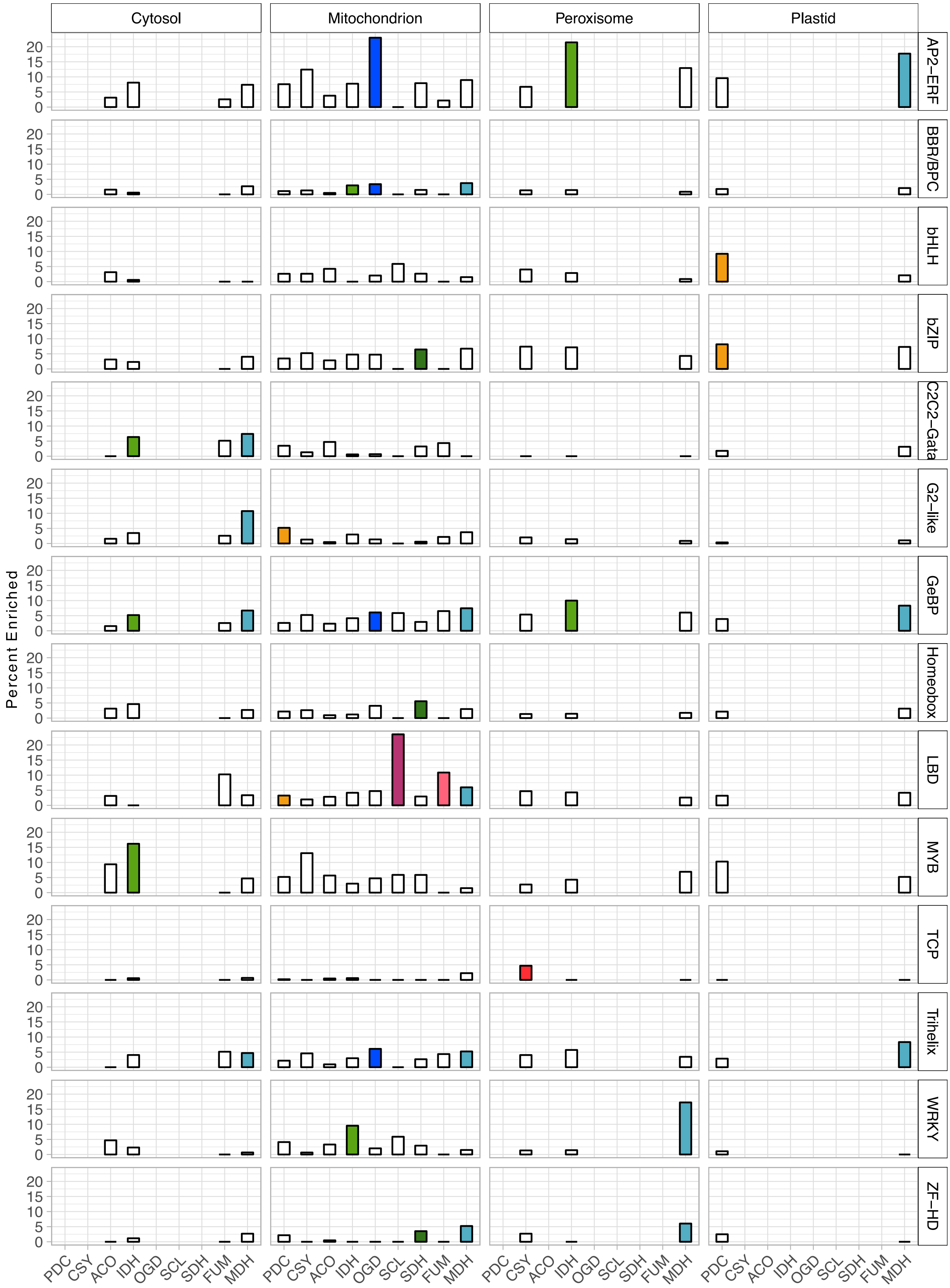

Figure EV2. Transcription factor (TF) families are enriched for tricarboxylic acid (TCA) cycle gene targets in specific cellular compartments.

Bar graphs display the percentage of TFs enriched for binding to promoters of the TCA cycle enzyme in the cytosol, mitochondrion, peroxisome, and plastid. The bar is colored if the TF family targeting the TCA cycle enzyme in the cellular compartment is significant (adjusted $P < 0.05$, Fisher's Exact Test, Dataset EV7). The colors of bars correspond to the TCA cycle enzyme in Figs 4 and EV1.

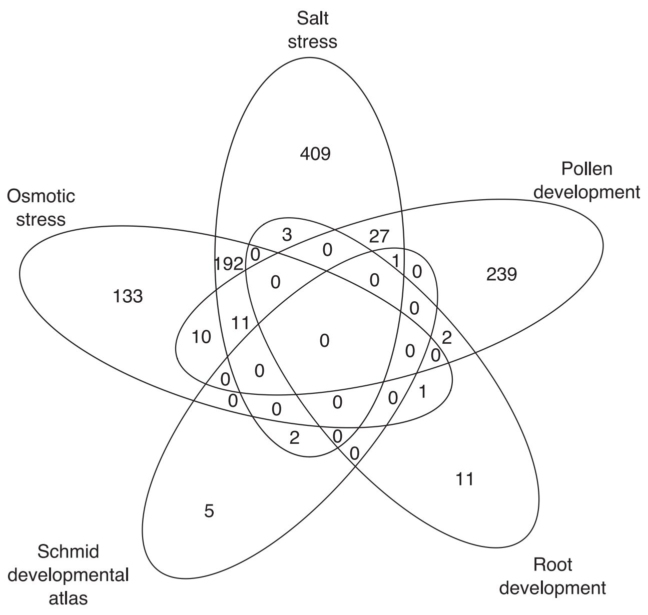

Figure EV3. Highly correlated transcription factor–tricarboxylic acid (TF–TCA) cycle target gene interactions shared between microarray datasets.

Numbers in the Venn diagram represent the TF–TCA cycle target gene co-expression with the absolute value of Pearson correlation coefficient ≥ 0.8 (Dataset EV8).

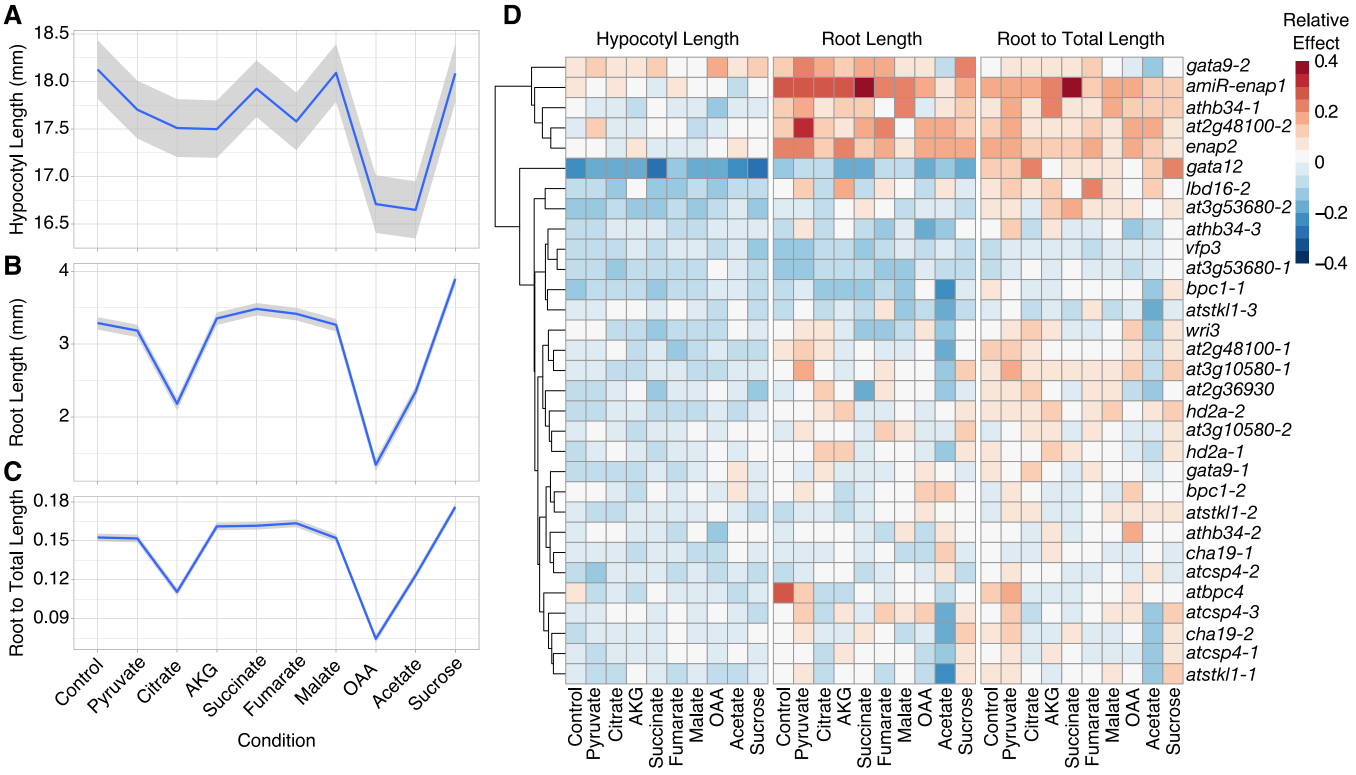

Figure EV4. Wild-type Col-0 and transcription factor (TF) mutant responses to tricarboxylic acid (TCA) metabolites.

A–C Hypocotyl length, root length, and the ratio of root to total length of *Arabidopsis thaliana* Col-0 seedlings grown on control or TCA metabolites. Data shown are mean (blue) ± SE (gray) calculated from ~200 seedlings per condition.

D Heat map summarizing the relative effect of TF mutant alleles on the hypocotyl length (left), root length (center), and the ratio of root to total length (right) on control or TCA metabolites-supplemented media. Mutant alleles are listed in rows.

© 2021 The Authors

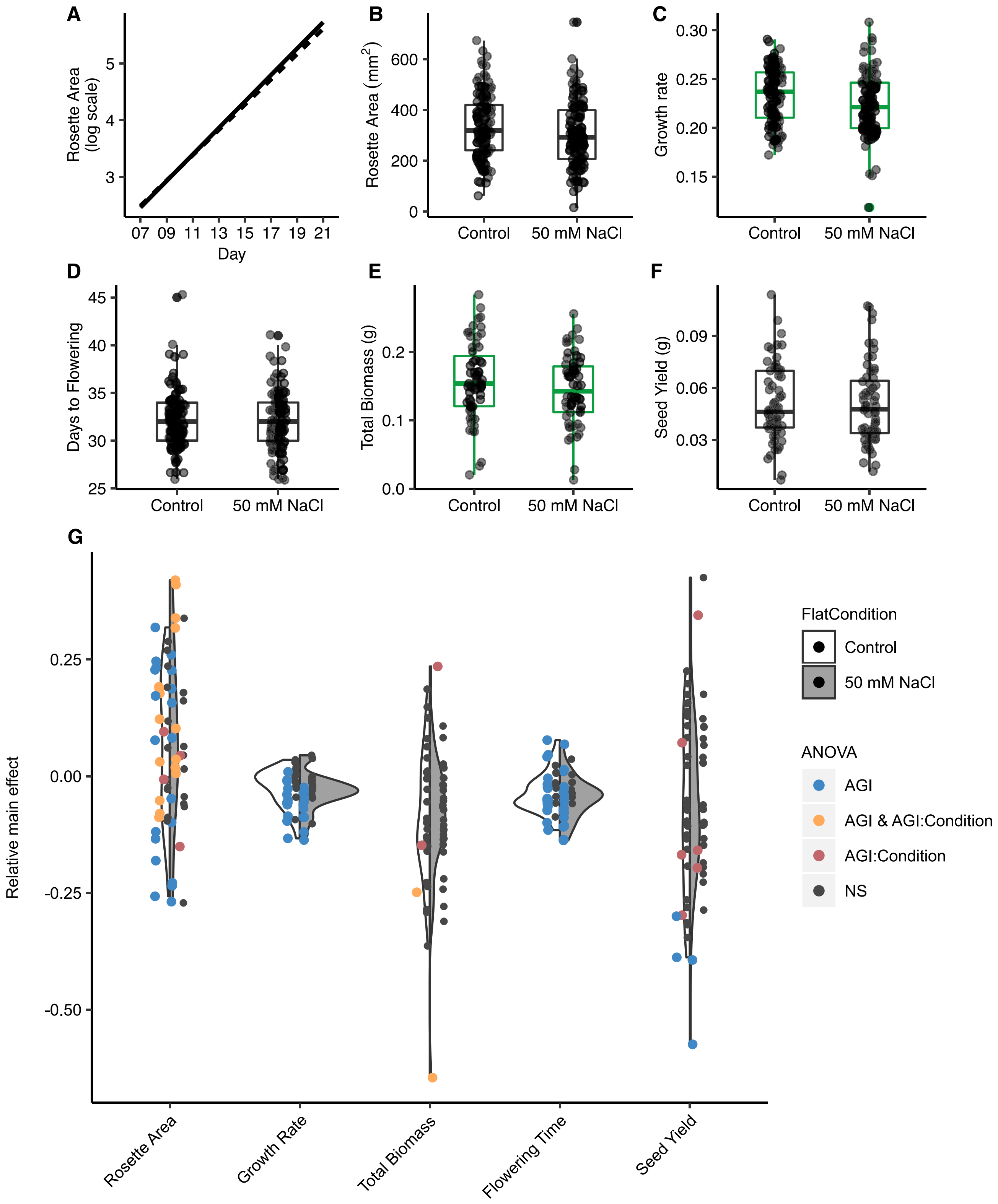
A
Rosette Area (log scale)
Day
B
Rosette Area (mm^2)
C
Growth rate
D
Days to Flowering
E
Total Biomass (g)
F
Seed Yield (g)
Control
50 mM NaCl
G
Relative main effect
Rosette Area
Growth Rate
Total Biomass
Flowering Time
Seed Yield
FlatCondition
Control
50 mM NaCl
ANOVA
AGI
AGI & AGI:Condition
AGI:Condition
NS

Figure EV5.

◀ **Figure EV5. Wild-type Col-0 and transcription factor (TF) mutant responses to salt treatment.**

A–F Rosette area, growth rate, days to flowering, dry shoot biomass, and seed yield of wild-type Col-0 under control and salt conditions. Solid line, control and dashed line, 50 mM NaCl. Green-colored box plots indicate significant differences between treatments ($P < 0.05$, Student's *t*-test, $N = 100$ plants per condition). Box plots mark the interquartile range, from the 25th to the 75th percentile, and are centered at the median. Whiskers extend to 1.5*interquartile range below the lower quartile and above the upper quartile.

G Distribution of relative effects of TF mutant alleles under control and salt stress conditions. Dots represent TF mutant alleles. TF mutant alleles are colored if the Arabidopsis Genome Initiative (AGI) (blue), AGI:Condition (red), and AGI and AGI:Condition (orange) linear model terms are significant ($P < 0.05$, two-way ANOVA). White violin, control condition; gray violin, salt stress condition.

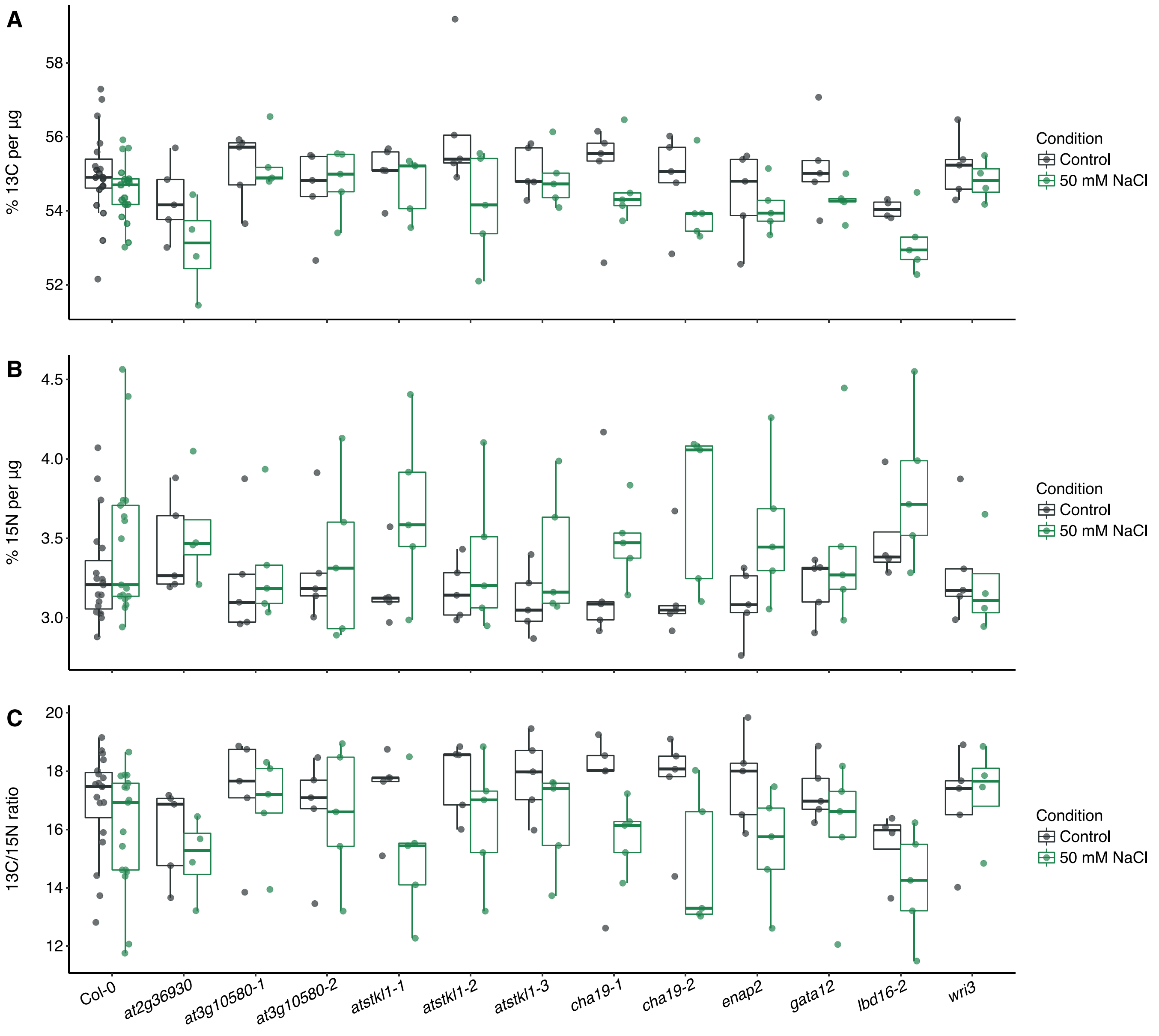

Figure EV6.

 © 2021 The Authors

◀ **Figure EV6. ^{13}Carbon, ^{15}nitrogen and the carbon:nitrogen ratio of transcription factor (TF) mutant alleles.**

A–C Percentages of ^{13}C, ^{15}N, and the ratio of ^{13}C:^{15}N of seeds of individuals grown in control and salt stress conditions. Box plots are shaded in if the Arabidopsis Genome Initiative (AGI) or AGI:Condition linear model terms were significant ($P < 0.05$, two-way ANOVA, N = 3–5 biological replicates for the mutant genotype per condition, 17 biological replicates for Col-0 per condition). Box plots mark the interquartile range, from the 25th to the 75th percentile, and are centered at the median. Whiskers extend to 1.5*interquartile range below the lower quartile and above the upper quartile. Individual measurements are plotted as dots.

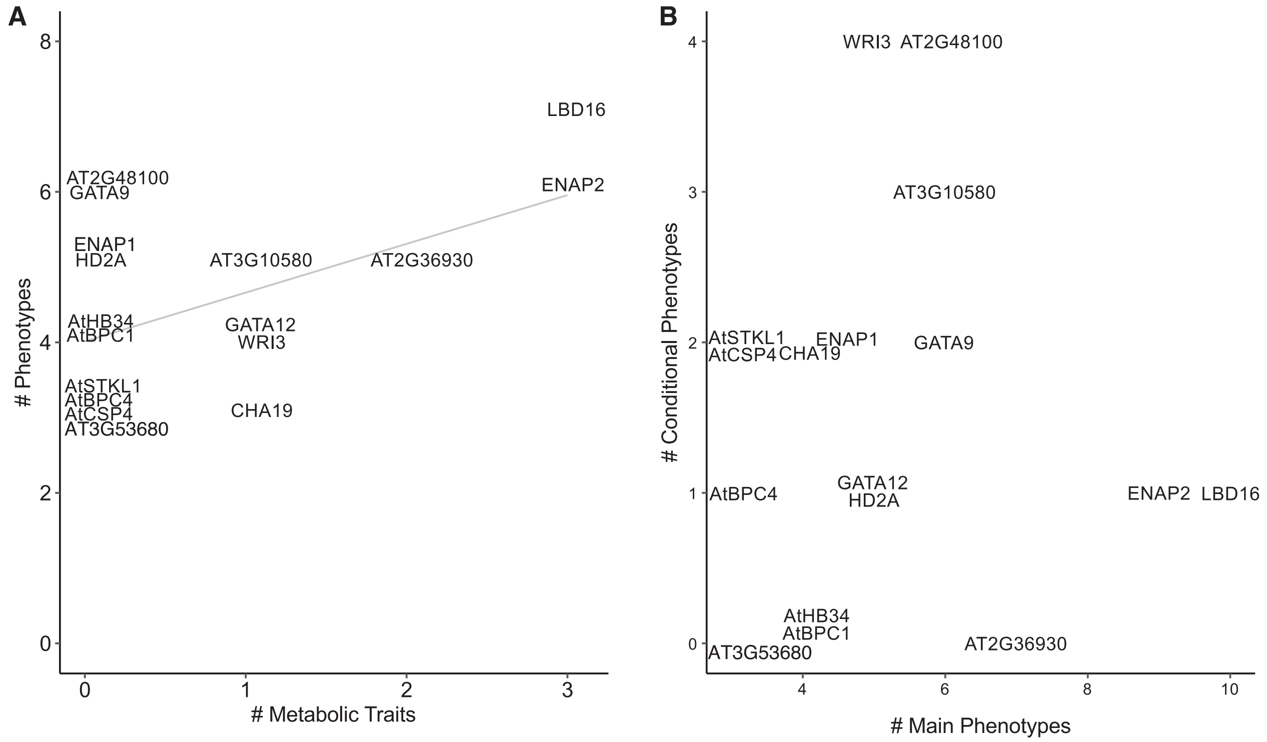

Figure EV7. Summary of transcription factor (TF) mutant growth traits and metabolic phenotypes.

A A positive correlation is observed between the number of significant growth phenotypes from the tricarboxylic acid (TCA) metabolite feeding and salt stress experiments and the number of significant metabolic phenotypes of seeds from the salt stress experiment (r = 0.53, R^2 = 0.28, P = 0.03, Pearson correlation).

B No correlation is observed between the total number of TFs with significant main effects and the total number of TFs with significant conditional (interaction) effects (r = 0.034, R^2 = 0.0011, P = 0.90, Pearson correlation).

© 2021 The Authors
