# Supplementary material for: A genome‐scale TF–DNA interaction network of transcriptional regulation of Arabidopsis primary and specialized metabolism
Source: Mol Syst Biol. 2021 Nov 24;17(11):e10625. doi: 10.15252/msb.202110625 (PMC8611409; doi:10.15252/msb.202110625)
Supplement: Supplementary file 15 [file MSB-17-e10625-s002.pdf]

molecular systems biology

A Genome-Scale Transcriptional Regulatory Network of Arabidopsis Primary and Specialized Metabolism

Siobhan Brady, Michelle Tang, Baohua Li, Xue Zhou, Tayah Bolt, Jia Jie Li, Neiman Cruz, Allison Gaudinier, Richard Ngo, Caitlin Clark-Wiest, and Daniel Kliebenstein
DOI: 10.15252/msb.202110625

Corresponding authors: Siobhan Brady (sbrady@ucdavis.edu) , Daniel Kliebenstein (Kliebenstein@ucdavis.edu)

Review Timeline:

Submission Date:	9th Aug 21
Editorial Decision:	20th Sep 21
Revision Received:	18th Oct 21
Editorial Decision:	25th Oct 21
Revision Received:	26th Oct 21
Accepted:	28th Oct 21

Editor: Maria Polychronidou

Transaction Report:

(Note: With the exception of the correction of typographical or spelling errors that could be a source of ambiguity, letters and reports are not edited. Depending on transfer agreements, referee reports obtained elsewhere may or may not be included in this compilation. Referee reports are anonymous unless the Referee chooses to sign their reports.)

RE: MSB-2021-10625, A Genome-Scale Transcriptional Regulatory Network of Arabidopsis Primary and Specialized Metabolism

Thank you again for submitting your work to Molecular Systems Biology. We have now heard back from the three referees who agreed to evaluate your study. Overall, the reviewers acknowledge that the study is a relevant contribution to the field. They raise however a series of concerns, which we would ask you to address in a revision.

I think that the reviewers' recommendations are clear and I therefore see no need to repeat the points listed below. All issues raised would need to be satisfactorily addressed. Please contact me in case you would like to discuss in further detail any of the issues raised.

Reviewer #1:

Summary
This manuscript "A Genome-Scale TF-DNA Interaction Network of Transcriptional Regulation of Arabidopsis Primary and Specialized Metabolism" by Tang et al. focuses on the construction of a TF-target network on genes involving in primary and specialized metabolism. The authors have used a global collection of Arabidopsis transcription factors (TFs) and 224 promoters pertinent to primary and specialized metabolism and perform a large-scale Y1H experiment. This led to the identification of 7,485 interactions between 1,930 TFs and 220 promoters across the 12 pathways. The authors chose four TFs CHROMATIN REMODELING 19 (CHA19), EIN2 NUCLEAR ASSOCIATED PROTEIN 1 (ENAP1), LATERAL ORGAN BOUNDARIES-DOMAIN 16 (LBD16) and WRINKLED 3 (WRI3) and tested their regulatory capacity. Using GR-TF method, they look for direct and indirect targets of these four TFs. Subsequently, the authors focused on TCA cycle and identified 17 TFs that can contribute to TCA-cycle dependent plant growth using reverse genetics approach. Specifically, they obtained 31 insertional mutant alleles of 17 TFs. Importantly, Of the seventeen tested TF and their corresponding mutant alleles, twelve TFs have significant genotype-dependent 306 hypocotyl growth effects. Next, the authors focused on salt stress. Mutations in fifteen of the TCA cycle-linked TFs led to small but significant effects on shoot biomass, flowering time, growth rate and seed yield in both control and salt conditions relative to wild-type seedlings

General remarks:
Overall, the manuscript addresses an essential process of biology using a systems biology approach. The experiments are performed unbiasedly to identify novel players in plant stress biology that are interconnected with plant metabolism. This manuscript is definitely a step forward in our understanding of an essential area of biology that is common to every plant species. The knowledge gained from this work can be extrapolated on important crop plants for better crop production as well as protection against other environmental stresses including light and salt.

Major Points:

The manuscript is well-thought-out and all experiments are executed with greater details. No major points are listed by this reviewer.

Minor Points:
I may have missed that did the authors compare their Y1H-based network vs co-expression network constructed from salt and light-dependent stresses? Such comparison would further substantiate the findings of this manuscript.

Reviewer #2:

This is a really nice study that demonstrates the principle of distributed control in biological networks. The focus on context-specific regulation of the TCA cycle is an interesting choice since TCA cycle intermediates contribute to the synthesis of proteins, lipids and other macromolecules, but also act in the regulation of chromatin modifications and post-translational modifications by contributing acetyl groups. Thus, the TCA cycle itself is considered a signaling hub, and is an indicator of cellular energy supplies.
The study itself lays out that there are a bunch of tfs that regulate many genes in many pathways, but not all tfs are regulating all the genes all the time. Some tfs will only regulate a pathway in a certain subcellular compartment, and some tfs will only regulate a pathway in response to a particular developmental or environmental cue. The salt stress analysis demonstrates this nicely. The dark-grown experiment is a little confusing though. With the hypothesis of context-specific regulation of the TCA cycle, and the absence of a universal set of TF/TCA target interactions, was it expected that the same 17 TFs would so strongly influence phenotypes under dark-grown and salt stress conditions? This seems in conflict with the distributed control idea.

Minor points:
Several times throughout the ms promoters are referred to in the context of metabolic pathways (eg: line 28 "promoters of primary and specialized metabolism" and line 115 "promoters of multiple metabolic pathways") rather than genes. It would be more precise to always say "gene promoters in primary and specialized metabolic pathways."

Using the subcellular localization to constrain TF-target interactions is a really nice idea. Did you also look at organ-specific TF expression (eFP browser) as part of the "context specific" information? Would root vs shoot expression have local effects on hypocotyl and root phenotypes, or do you think the phenotypes are the result of global changes in TCA cycle derived metabolites?

A small issue with the TCA cycle-focused part: since the above sections demonstrate that single TFs regulate several genes across several pathways, how can the observed changes in plant growth, development, and response to the environment to TF perturbation be specifically linked to changes in the TCA cycle without measuring the organic acids in the TCA cycle (as validation of direct impact), or without examining changes in expression in genes in the other affected pathways? For example wri3: 8 gene promoters in glycolysis/gluconeogenesis and the 3 gene promoters in PPP.

Line 276: extra word in the sentence.

Figure 5C and EV4D are showing the same thing, but the relative effect scale is different. 5C makes the wri3 result look much more significant than it does in EV4D. It just looks less sketchy if they're the same.

Lines 386-388 state that the authors propose that plant metabolism is controlled via a distributed system... large collections of TFs (similar statement in lines 405-407). That's kinda the central thesis of biological networks that biological systems are dominated by distributed control (very few "hub" genes), so this research provides support for that rather than being a novel proposed mechanism.

Reviewer #3:

Summary
The study aims to systematically characterize the complexity of metabolic networks from the perspective of transcriptional regulation in multicellular organisms. Specifically, the authors hypothesized that Arabidopsis metabolism is orchestrated by TFs that regulate both primary and specialized metabolism.

Although Y1H suffers from false positive and false negatives, the author performed Y1H screening between 1930 TFs and 220 promoters across 11 central carbon metabolism pathways and one specialized metabolism pathway (aliphatic glucosinolate biosynthesis). The authors revealed that 90% of TFs bind to promoters of metabolic genes and all TFs bind to promoters of multiple metabolic pathways.

Then the authors tested the regulatory capacity of 4 selected TFs by performing transcriptome analyses of DEX-inducible lines of 4TFs. The results showed that the predicted target enzyme genes were enriched among the DEGs in the respective DEX-

inducible lines.

The authors focused on TCA cycle isozymes and showed the enrichment of certain TF families in isozymes localized to specific cellular compartments. Coexpression relationship between TF and TCA cycle isozyme genes across 5 microarray datasets supported the idea that regulation of TCA cycle genes is highly conditional.

The phenotypic analyses of mutant alleles of 17 TFs within the TCA cycle sub-network
demonstrated the importance of the regulatory module in plant growth and its response to salt stress as well as the importance of TFs in coordination of TCA cycle function in different organs and conditions.

General remarks
In the case of primary metabolism in plant, transcriptional regulation is not obvious compared to specialized secondary metabolism in which a relatively small number of TFs regulate the most part of enzyme genes involved. This study clarified interaction of TFs and promoters of central carbon metabolism genes (and aliphatic glucosinolate biosynthetic genes) in a genome-scale. It is surprising that an average of 125 transcription factors are bound to one promoter. The authors performed well-designed phenotypic analyses focusing of sub-network of TCA cycle regulation to validate the finding from Y1H. This study provides useful datasets to understand complicated regulatory mechanisms of primary and specialized metabolism.

Minor comments
- Please carefully check the numbers of promoters and TFs used.
For example, line130 says "224 promoters", while 203 genes (except 22 aliphatic glucosinolate genes) are listed in Table EV1. Similarly, line138 says "additional 1227 TFs", while 1225 TF genes are listed in TableEV2.

- line167-177
If I understand it correctly, regional enrichment can be clarified by a hypergeometric test. I missed concrete description of the method and result of this test.

- line203-217
I got a bit confused in understanding of Fig.3C-E. First of all, Panel C and Panel D seem reversed.
"Gene ontologies associated with the reductive pentose phosphate pathway, glycine biosynthesis, and cysteine biosynthesis were over-represented more than expected by chance in the significantly up-regulated DEGs upon GR induction of CHA19, LBD16 and WRI3". I could not find such a result in Panel D. There is no GO terms "glycine biosynthesis" and "cysteine biosynthesis" in the figure. "reductive pentose phosphate pathway" does not seem enriched in GR-LBD16.
More detailed explanation is better to be added in Fig.3E legend.

- line266
I think "TF/TCA target interaction" is better to be "TF/TCA target coexpression" to avoid readers' confusion. "interaction" is also used to explain TF-promoter binding in Y1H.

- line281-282 "root length of the wri3 loss-of-function mutant allele is shorter compared to wild type (Fig 5C-E)."
In Fig.5E, the root length of wri3 looks longer than that of WT in control condition.

- line316-317
(Fig.5C) may be (Fig.5D).

- line346-347 "BASIC PENTACYSTEINE 4, COLD SHOCK DOMAIN PROTEIN 4"
Please add abbreviations as shown in FIg.6A.

-line618 "rifampin" may be "refampicin".

- On which database did gene annotation depend?

- Fig.1 Aliphatic glucosinolate pathway was not found in Panel A.

- In Fig.5C, the relative effect of atbpc4 on root length is almost zero, while that in Fig EV4 is around 0.4. Why?

- Fig.5(A) legend
"Arabidopsis thaliana" should be italicized.

- Fig.6 legend
Please add the legend for Panel B. Accordingly, (B) and (C) in the legend should be (C) and (D). "Solid lines, Col-0; dashed line, bpc4; circle, control condition; triangle, 50 mM NaCl." (line764) should be moved to the legend for Panel C.
In Panel A, "Total Biomass" is better to be "Shoot Biomass".

- Fig.EV3 legend
TF-TCA target gene "interactions" is better to be "coexpression".

- English proofreading may be recommended for some points.

- Table EV3
For example, MYB28 bound to a limited number of aliphatic GSL genes. I wonder why MYB28 did not bind to CYP79F1, MAM1, etc.

- Table EV5
More detailed explanation is desirable.

Reviewer #1:

Summary
This manuscript "A Genome-Scale TF-DNA Interaction Network of Transcriptional Regulation of Arabidopsis Primary and Specialized Metabolism" by Tang et al. focuses on the construction of a TF-target network on genes involving in primary and specialized metabolism. The authors have used a global collection of Arabidopsis transcription factors (TFs) and 224 promoters pertinent to primary and specialized metabolism and perform a large-scale Y1H experiment. This led to the identification of 7,485 interactions between 1,930 TFs and 220 promoters across the 12 pathways. The authors chose four TFs CHROMATIN REMODELING 19 (CHA19), EIN2 NUCLEAR ASSOCIATED PROTEIN 1 (ENAP1), LATERAL ORGAN BOUNDARIES-DOMAIN 16 (LBD16) and WRINKLED 3 (WRI3) and tested their regulatory capacity. Using GR-TF method, they look for direct and indirect targets of these four TFs. Subsequently, the authors focused on TCA cycle and identified 17 TFs that can contribute to TCA-cycle dependent plant growth using reverse genetics approach. Specifically, they obtained 31 insertional mutant alleles of 17 TFs. Importantly, Of the seventeen tested TF and their corresponding mutant alleles, twelve TFs have significant genotype-dependent 306 hypocotyl growth effects. Next, the authors focused on salt stress. Mutations in fifteen of the TCA cycle-linked TFs led to small but significant effects on shoot biomass, flowering time, growth rate and seed yield in both control and salt conditions relative to wild-type seedlings

General remarks:
Overall, the manuscript addresses an essential process of biology using a systems biology approach. The experiments are performed unbiasedly to identify novel players in plant stress biology that are interconnected with plant metabolism. This manuscript is definitely a step forward in our understanding of an essential area of biology that is common to every plant species. The knowledge gained from this work can be extrapolated on important crop plants for better crop production as well as protection against other environmental stresses including light and salt.

Major Points:
The manuscript is well-thought-out and all experiments are executed with greater details. No major points are listed by this reviewer.

Thank you for your positive comments!

Minor Points:
I may have missed that did the authors compare their Y1H-based network vs co-expression network constructed from salt and light-dependent stresses? Such comparison would further substantiate the findings of this manuscript.

We calculated co-expression values for TFs and their target genes using connections predicted by the Y1H network. The results of this analysis are summarized in Dataset EV8. The reviewer is correct that this comparison further substantiated the findings from the Y1H network. We then chose TFs that were strongly co-expressed with their target genes for further functional analysis.

Reviewer #2:

This is a really nice study that demonstrates the principle of distributed control in biological networks. The focus on context-specific regulation of the TCA cycle is an interesting choice

since TCA cycle intermediates contribute to the synthesis of proteins, lipids and other macromolecules, but also act in the regulation of chromatin modifications and post-translational modifications by contributing acetyl groups. Thus, the TCA cycle itself is considered a signaling hub, and is an indicator of cellular energy supplies.
The study itself lays out that there are a bunch of tfs that regulate many genes in many pathways, but not all tfs are regulating all the genes all the time. Some tfs will only regulate a pathway in a certain subcellular compartment, and some tfs will only regulate a pathway in response to a particular developmental or environmental cue. The salt stress analysis demonstrates this nicely.

The dark-grown experiment is a little confusing though. With the hypothesis of context-specific regulation of the TCA cycle, and the absence of a universal set of TF/TCA target interactions, was it expected that the same 17 TFs would so strongly influence phenotypes under dark-grown and salt stress conditions? This seems in conflict with the distributed control idea.

We had some inclination that these 17 TFs would affect the phenotypes under the conditions we tested due to the co-expression analysis results utilizing cell-/tissue-specific and abiotic stress expression datasets (Dataset EV8). We consider our results in agreement with the distributed control idea since these 17 TFs had significant but small effects on the phenotypes we measured indicating that no single TF had large effects on the higher-order phenotypes (Dataset EV10-12).

Minor points:
Several times throughout the ms promoters are referred to in the context of metabolic pathways (eg: line 28 "promoters of primary and specialized metabolism" and line 115 "promoters of multiple metabolic pathways") rather than genes. It would be more precise to always say "gene promoters in primary and specialized metabolic pathways."

References to promoters are updated to say “gene promoters in . . . metabolic pathways” or “promoters of genes in . . . pathways” in lines 28, 119, 162, 168, 272, 418.

Using the subcellular localization to constrain TF-target interactions is a really nice idea. Did you also look at organ-specific TF expression (eFP browser) as part of the "context specific" information? Would root vs shoot expression have local effects on hypocotyl and root phenotypes, or do you think the phenotypes are the result of global changes in TCA cycle derived metabolites?

We thank the reviewer for this suggestion. Although datasets profiling expression in both the root and the shoot were included in our co-expression analyses for TFs and targets, we did not explicitly choose TFs for validation based on their organ-specific TF expression. Looking at the hypocotyl and root expression of the TFs we had biologically validated on the eFP browser, we did not see any obvious similarities between organ-specific expression pattern and whether the mutants of the TFs will significantly influence the traits we measured. We saw significant effects in hypocotyl or root when the expression of TFs were both high or below the limit of detection (likely indicating very low expression or specific spatiotemporal expression not captured by whole organ profiling).
TF expression levels were similar in the hypocotyl and in the root for the majority of the TFs we functionally validated. An exception to this is for the cases where TF expression was higher in one of the organs for *GATA9* and *GATA12*, hypocotyl and root phenotypes, respectively, were significantly influenced by the TF.

A small issue with the TCA cycle-focused part: since the above sections demonstrate that single TFs regulate several genes across several pathways, how can the observed changes in plant growth, development, and response to the environment to TF perturbation be specifically linked to changes in the TCA cycle without measuring the organic acids in the TCA cycle (as validation of direct impact), or without examining changes in expression in genes in the other affected pathways? For example wri3: 8 gene promoters in glycolysis/gluconeogenesis and the 3 gene promoters in PPP.

As suggested by the reviewer, we had measured the expression changes in genes in other pathways in GR-CHA19, GR-ENAP1, GR-LBD16 and GR-WRI3 lines and that data can be found in Fig 3 and Dataset EV6.
We appreciate the reviewer's suggestion about measuring the organic acids in the TCA cycle, however the interpretation of direct causality is complicated given the anaplerotic nature of the TCA cycle. This leads to the potential for steady state levels of the metabolite and the fluxes to be significantly disconnected and the organellar and even cellular specific expression patterns of these TFs and the target genes indicate that it isn't clear what specific metabolic measurements would be appropriate. Further, given the connection of the TCA cycle to a wide range of metabolites, it isn't clear if the organic acid changes themselves would be expected to be directly causal. We feel that this question is critical for future studies where it would need a combination of steady state and flux-based measurements on organs if not single cells to map causality. And even then there may not be single causalities as for example, if the TCA cycle alters the shikimate pathway, this could lead to both auxin and lignin alterations. This complexity is the reason we instead focused on growth based assays to try and capture the higher-order consequences of changes in the CHA19, ENAP1, LBD16 and WRI3 TFs that bind to promoters of genes in multiple metabolic pathways.

Line 276: extra word in the sentence.

The extra word (currently) in line 301 has been removed.

Figure 5C and EV4D are showing the same thing, but the relative effect scale is different. 5C makes the wri3 result look much more significant than it does in EV4D. It just looks less sketchy if they're the same.

While the data underlying Figure 5C and Figure EV4D are the same, the plots are showing different aspects of the results. Figure 5C is a summary heatmap, showing the averages of all the effects in Figure EV4D) whereas Figure EV4D displays the effects of the mutants under each of the conditions tested. The scales are set based on the maximum and minimum relative effect for each plot, which are different between the two plots.

Lines 386-388 state that the authors propose that plant metabolism is controlled via a distributed system... large collections of TFs (similar statement in lines 405-407). That's kinda the central thesis of biological networks that biological systems are dominated by distributed control (very few "hub" genes), so this research provides support for that rather than being a novel proposed mechanism.

Reviewer #3:

Summary
The study aims to systematically characterize the complexity of metabolic networks from the perspective of transcriptional regulation in multicellular organisms. Specifically, the authors hypothesized that Arabidopsis metabolism is orchestrated by TFs that regulate both primary and specialized metabolism.

Although Y1H suffers from false positive and false negatives, the author performed Y1H screening between 1930 TFs and 220 promoters across 11 central carbon metabolism pathways and one specialized metabolism pathway (aliphatic glucosinolate biosynthesis). The authors revealed that 90% of TFs bind to promoters of metabolic genes and all TFs bind to promoters of multiple metabolic pathways.

Then the authors tested the regulatory capacity of 4 selected TFs by performing transcriptome analyses of DEX-inducible lines of 4TFs. The results showed that the predicted target enzyme genes were enriched among the DEGs in the respective DEX-inducible lines.

The authors focused on TCA cycle isozymes and showed the enrichment of certain TF families in isozymes localized to specific cellular compartments. Coexpression relationship between TF and TCA cycle isozyme genes across 5 microarray datasets supported the idea that regulation of TCA cycle genes is highly conditional.

The phenotypic analyses of mutant alleles of 17 TFs within the TCA cycle sub-network demonstrated the importance of the regulatory module in plant growth and its response to salt stress as well as the importance of TFs in coordination of TCA cycle function in different organs and conditions.

General remarks
In the case of primary metabolism in plant, transcriptional regulation is not obvious compared to specialized secondary metabolism in which a relatively small number of TFs regulate the most part of enzyme genes involved. This study clarified interaction of TFs and promoters of central carbon metabolism genes (and aliphatic glucosinolate biosynthetic genes) in a genome-scale. It is surprising that an average of 125 transcription factors are bound to one promoter. The authors performed well-designed phenotypic analyses focusing of sub-network of TCA cycle regulation to validate the finding from Y1H. This study provides useful datasets to understand complicated regulatory mechanisms of primary and specialized metabolism.

Minor comments
- Please carefully check the numbers of promoters and TFs used.
For example, line130 says "224 promoters", while 203 genes (except 22 aliphatic glucosinolate genes) are listed in Table EV1. Similarly, line138 says "additional 1227 TFs", while 1225 TF genes are listed in TableEV2.

We thank the reviewer for their careful review of our data. Table EV1 summarizes AGIs, gene names, promoter lengths and cloning primers for 203 genes. One of the gene promoters, promoter of AT3G54050, was cloned with different primers and subsequently screened. We included both versions and the resulting Y1H contains TFs detected by both bait strains. We also corrected these numbers in lines 133 and 143 to reflect the number in Table EV2 (1224 TFs + one duplicate).

- line167-177
If I understand it correctly, regional enrichment can be clarified by a hypergeometric test. I missed concrete description of the method and result of this test.

This summary of how aliphatic GSL is integrated with central carbon metabolism is an extension of the previous paragraph. The method involves calculating the significance of association between two pathways, for all pairwise combinations of pathways, followed by ordered ranking of the adjusted p-values of the Fisher's exact test (hypergeometric distribution) to determine the metabolic pathways that share the most TFs with aliphatic GSL. We have added a reference to the result table (Dataset EV4) as well included a description of our methods in the section "Pairwise Pathway Comparison" (lines 548 to 556).

- line203-217
I got a bit confused in understanding of Fig.3C-E. First of all, Panel C and Panel D seem reversed.
"Gene ontologies associated with the reductive pentose phosphate pathway, glycine biosynthesis, and cysteine biosynthesis were over-represented more than expected by chance in the significantly up-regulated DEGs upon GR induction of CHA19, LBD16 and WRI3". I could not find such a result in Panel D. There is no GO terms "glycine biosynthesis" and "cysteine biosynthesis" in the figure. "reductive pentose phosphate pathway" does not seem enriched in GR-LBD16.
More detailed explanation is better to be added in Fig.3E legend.

We have updated Panel C and Panel D to match the figure legend and to focus on GO terms related to metabolism. We have edited the manuscript text to call out explicitly the enriched GO terms for each GR-TF in line 217 to 222. We included more detailed explanation for Fig. 3E legend (line 770 to 775).

- line266
I think "TF/TCA target interaction" is better to be "TF/TCA target coexpression" to avoid readers' confusion. "interaction" is also used to explain TF-promoter binding in Y1H.

We edited the text to clarify that we are describing both TF-promoter binding interactions and co-expression (line 286 to 287).

- line281-282 "root length of the wri3 loss-of-function mutant allele is shorter compared to wild type (Fig 5C-E)."
In Fig.5E, the root length of wri3 looks longer than that of WT in control condition.

Fig 5C shows the average of the *<u>relative effect</u>* of the *wri3* mutation across the three traits tested, while Fig 5E is not normalized to show relative effect, and instead shows the difference for <u>root length only</u> compared to wild type.

- line316-317
(Fig.5C) may be (Fig.5D).

The text has been revised to call out the correct figure panels (Fig 5B & 5D) (line 345).

- line346-347 "BASIC PENTACYSTEINE 4, COLD SHOCK DOMAIN PROTEIN 4"
Please add abbreviations as shown in FIg.6A.

Abbreviations of those genes as shown in Fig. 6A have been added (line 377 to 379).

-line618 "rifampin" may be "refampicin".

This error has been corrected to "rifampicin" in line 670.

- On which database did gene annotation depend?

The methods section, Gene Ontology Enrichment Analysis, has been updated to include version of gene annotation database (2019-10-08) (see lines 594 to 595).

- Fig.1 Aliphatic glucosinolate pathway was not found in Panel A.

Aliphatic glucosinolate (Aliphatic GSL) has been added to Fig 1A.

- In Fig.5C, the relative effect of atbpc4 on root length is almost zero, while that in Fig EV4 is around 0.4. Why?

Fig 5C shows the <u>averages of the relative effect</u> across all the conditions for each trait measured, while Fig EV4 shows the comparison to wild type for each condition where the wild type measurement is considered 0.

- Fig.5(A) legend
"Arabidopsis thaliana" should be italicized.

"Arabidopsis thaliana" is now italicized as listed in line 798.

- Fig.6 legend
Please add the legend for Panel B. Accordingly, (B) and (C) in the legend should be (C) and (D). "Solid lines, Col-0; dashed line, bpc4; circle, control condition; triangle, 50 mM NaCl." (line764) should be moved to the legend for Panel C.
In Panel A, "Total Biomass" is better to be "Shoot Biomass".

The legend for Fig 6 has been revised according to reviewer's recommendation. The legend for panel B has been added. Legend letters (C) and (D) have been updated to call out the correct panels. "Total Biomass" in panel A has been changed to "Shoot Biomass" (see lines 816-834).

- Fig.EV3 legend
TF-TCA target gene "interactions" is better to be "coexpression".

Text has been changed to "co-expression" in line 861.

- English proofreading may be recommended for some points.

The co-corresponding authors have reviewed the text for remaining grammatical errors.

- Table EV3
For example, MYB28 bound to a limited number of aliphatic GSL genes. I wonder why MYB28 did not bind to CYP79F1, MAM1, etc.

As the reviewer points out, in other in planta assays published by several groups, MYB28 does bind most of these promoters. Our current thoughts about why MYB28 seems to have a false negative issue in Y1H is that MYB28 may require bHLHs like MYC2, AP2/ERFs like RAP2.2 or a complex of these TFs to maximize its binding strength. This would be akin to the flavonoid and anthocyanin MYBs that use a tripartite TF complex to maximize binding.

- Table EV5
More detailed explanation is desirable.

The legend for table EV5 (now Dataset EV5) is edited to contain more details. The legend can be found in the Extended Dataset file.

Manuscript Number: MSB-2021-10625R, A Genome-Scale Transcriptional Regulatory Network of Arabidopsis Primary and Specialized Metabolism

Thank you for sending us your revised manuscript. We think that the performed revisions have satisfactorily addressed the issues raised by the reviewers. As such, I am glad to inform you that we can soon proceed with formally accepting the study for publication, pending some minor editorial issues listed below. We would ask you to address these issues in a minor revision.

The authors performed the requested editorial changes.

RE: MSB-2021-10625RR, A Genome-Scale Transcriptional Regulatory Network of Arabidopsis Primary and Specialized Metabolism

Thank you again for sending us your revised manuscript. We are now satisfied with the modifications made and I am pleased to inform you that your paper has been accepted for publication.

EMBO PRESS

YOU MUST COMPLETE ALL CELLS WITH A PINK BACKGROUND ↓

PLEASE NOTE THAT THIS CHECKLIST WILL BE PUBLISHED ALONGSIDE YOUR PAPER

Corresponding Author Name: Siobhan M. Brady

Journal Submitted to: Molecular Systems Biology

Manuscript Number: MSB-2021-10625

Reporting Checklist For Life Sciences Articles (Rev. June 2017)

This checklist is used to ensure good reporting standards and to improve the reproducibility of published results. These guidelines are consistent with the Principles and Guidelines for Reporting Preclinical Research issued by the NIH in 2014. Please follow the journal's authorship guidelines in preparing your manuscript.

A- Figures
1. Data
The data shown in figures should satisfy the following conditions:

- the data were obtained and processed according to the field's best practice and are presented to reflect the results of the experiments in an accurate and unbiased manner.
- figure panels include only data points, measurements or observations that can be compared to each other in a scientifically meaningful way.
- graphs include clearly labeled error bars for independent experiments and sample sizes. Unless justified, error bars should not be shown for technical replicates.
- if n< 5, the individual data points from each experiment should be plotted and any statistical test employed should be justified
- Source Data should be included to report the data underlying graphs. Please follow the guidelines set out in the author ship guidelines on Data Presentation.

2. Captions

Each figure caption should contain the following information, for each panel where they are relevant:

- a specification of the experimental system investigated (eg cell line, species name).
- the assay(s) and method(s) used to carry out the reported observations and measurements
- an explicit mention of the biological and chemical entity(ies) that are being measured.
- an explicit mention of the biological and chemical entity(ies) that are altered/varied/perturbed in a controlled manner.

- the exact sample size (n) for each experimental group/condition, given as a number, not a range;
- a description of the sample collection allowing the reader to understand whether the samples represent technical or biological replicates (including how many animals, litters, cultures, etc.).
- a statement of how many times the experiment shown was independently replicated in the laboratory.
- definitions of statistical methods and measures:
  - common tests, such as t-test (please specify whether paired vs. unpaired), simple χ2 tests, Wilcoxon and Mann-Whitney tests, can be unambiguously identified by name only, but more complex techniques should be described in the methods section;
  - are tests one-sided or two-sided?
  - are there adjustments for multiple comparisons?
  - exact statistical test results, e.g., P values = x but not P values < x;
  - definition of 'center values' as median or average;
  - definition of error bars as s.d. or s.e.m.

Any descriptions too long for the figure legend should be included in the methods section and/or with the source data.

In the pink boxes below, please ensure that the answers to the following questions are reported in the manuscript itself. Every question should be answered. If the question is not relevant to your research, please write NA (non applicable). We encourage you to include a specific subsection in the methods section for statistics, reagents, animal models and human subjects.

USEFUL LINKS FOR COMPLETING THIS FORM

http://www.antibodypedia.com
http://1degreebio.org
http://www.equator-network.org/reporting-guidelines/improving-bioscience-research-repor
http://grants.nih.gov/grants/olaw/olaw.htm
http://www.mrc.ac.uk/Ourresearch/Ethicsresearchguidance/Useofanimals/index.htm
http://ClinicalTrials.gov
http://www.consort-statement.org
http://www.consort-statement.org/checklists/view/32-consort/66-title
http://www.equator-network.org/reporting-guidelines/reporting-recommendations-for-tumo
http://datadryad.org
http://figshare.com
http://www.ncbi.nlm.nih.gov/gap
http://www.ebi.ac.uk/ega
http://biomodels.net/
http://biomodels.net/miriam/
http://jjj.biochem.sun.ac.za
https://osp.od.nih.gov/biosafety-biosecurity-and-emerging-biotechnology/
http://www.selectagents.gov/

B- Statistics and general methods

Please fill out these boxes ↓ (Do not worry if you cannot see all your text once you press return)

1.a. How was the sample size chosen to ensure adequate power to detect a pre-specified effect size?	Replication numbers for all experiments were designed to provide significant power for moderate effect sizes and modest power for small effect sizes using a presumed broad-sense heritability of about 15% based on previous publications and data for these traits.
1.b. For animal studies, include a statement about sample size estimate even if no statistical methods were used.	NA
2. Describe inclusion/exclusion criteria if samples or animals were excluded from the analysis. Were the criteria pre-established?	Any plants that did not survive the full duration of phenotyping assay were excluded from analysis. Two 35S:GR-WRI3 RNA-Seq libraries, L8_dex_rep1 and L10_mock_rep2, were excluded from downstream analysis due to being outliers in the principal component analysis of normalized read counts.
3. Were any steps taken to minimize the effects of subjective bias when allocating animals/samples to treatment (e.g. randomization procedure)? If yes, please describe.	Mutant lines were assigned random aliases for experiments and analysis. Gene mutant names were linked to the data after the analysis.
For animal studies, include a statement about randomization even if no randomization was used.	NA
4.a. Were any steps taken to minimize the effects of subjective bias during group allocation or/and when assessing results (e.g. blinding of the investigator)? If yes please describe.	Phenotyping assays were performed in a semi-random block design. For both the TCA feeding and the salt response assays, each plate/flat had a wild type Col-0 control to which each mutant was compared. Samples for RNA-Seq were plated on individual plates and placed in a random order in the dark growth chamber. After mock or Dex treatment, plates were placed in the same random order as before treatment.
4.b. For animal studies, include a statement about blinding even if no blinding was done	NA
5. For every figure, are statistical tests justified as appropriate?	Yes
Do the data meet the assumptions of the tests (e.g., normal distribution)? Describe any methods used to assess it.	We used generalized linear mixed models when determining statistical significance in response variables between groups because generalized linear mixed models allow for more flexibility in the data, particularly when distribution of data deviates from normal and variance is not constant between groups.

Is there an estimate of variation within each group of data?	Yes, variance is provided in expanded tables.
Is the variance similar between the groups that are being statistically compared?	No, hence we used generalized linear mixed models when determining statistical significance in response variables between groups because generalized linear mixed models allow for more flexibility in the data, particularly when distribution of data deviates from normal and variance is not constant between groups.

C- Reagents

6. To show that antibodies were profiled for use in the system under study (assay and species), provide a citation, catalog number and/or clone number, supplementary information or reference to an antibody validation profile. e.g., Antibodypedia (see link list at top right), 1DegreeBio (see link list at top right).	NA
7. Identify the source of cell lines and report if they were recently authenticated (e.g., by STR profiling) and tested for mycoplasma contamination.	NA

* for all hyperlinks, please see the table at the top right of the document

D- Animal Models

8. Report species, strain, gender, age of animals and genetic modification status where applicable. Please detail housing and husbandry conditions and the source of animals.	NA
9. For experiments involving live vertebrates, include a statement of compliance with ethical regulations and identify the committee(s) approving the experiments.	NA
10. We recommend consulting the ARRIVE guidelines (see link list at top right) (PLoS Biol. 8(6), e1000412, 2010) to ensure that other relevant aspects of animal studies are adequately reported. See author guidelines, under 'Reporting Guidelines'. See also: NIH (see link list at top right) and MRC (see link list at top right) recommendations. Please confirm compliance.	NA

E- Human Subjects

11. Identify the committee(s) approving the study protocol.	NA
12. Include a statement confirming that informed consent was obtained from all subjects and that the experiments conformed to the principles set out in the WMA Declaration of Helsinki and the Department of Health and Human Services Belmont Report.	NA
13. For publication of patient photos, include a statement confirming that consent to publish was obtained.	NA
14. Report any restrictions on the availability (and/or on the use) of human data or samples.	NA
15. Report the clinical trial registration number (at ClinicalTrials.gov or equivalent), where applicable.	NA
16. For phase II and III randomized controlled trials, please refer to the CONSORT flow diagram (see link list at top right) and submit the CONSORT checklist (see link list at top right) with your submission. See author guidelines, under 'Reporting Guidelines'. Please confirm you have submitted this list.	NA
17. For tumor marker prognostic studies, we recommend that you follow the REMARK reporting guidelines (see link list at top right). See author guidelines, under 'Reporting Guidelines'. Please confirm you have followed these guidelines.	NA

F- Data Accessibility

18: Provide a "Data Availability" section at the end of the Materials & Methods, listing the accession codes for data generated in this study and deposited in a public database (e.g. RNA-Seq data: Gene Expression Omnibus GSE39462, Proteomics data: PRIDE PXD000208 etc.) Please refer to our author guidelines for 'Data Deposition'.<br><br>Data deposition in a public repository is mandatory for:<br>a. Protein, DNA and RNA sequences<br>b. Macromolecular structures<br>c. Crystallographic data for small molecules<br>d. Functional genomics data<br>e. Proteomics and molecular interactions	The datasets and computer code produced in this study are available in the following databases:<br>• RNA-Seq data: Gene Expression Omnibus GSE137623: https://www.ncbi.nlm.nih.gov/geo/query/acc.cgi?acc=GSE137623.<br>• Raw data files and analysis scripts: GitHub: https://github.com/melletang/ccp_y1h
19. Deposition is strongly recommended for any datasets that are central and integral to the study; please consider the journal's data policy. If no structured public repository exists for a given data type, we encourage the provision of datasets in the manuscript as a Supplementary Document (see author guidelines under 'Expanded View' or in unstructured repositories such as Dryad (see link list at top right) or Figshare (see link list at top right).	Datasets will provided as Expanded View Datasets or specified in the Data Availability section.
20. Access to human clinical and genomic datasets should be provided with as few restrictions as possible while respecting ethical obligations to the patients and relevant medical and legal issues. If practically possible and compatible with the individual consent agreement used in the study, such data should be deposited in one of the major public access-controlled repositories such as dbGAP (see link list at top right) or EGA (see link list at top right).	NA
21. Computational models that are central and integral to a study should be shared without restrictions and provided in a machine-readable form. The relevant accession numbers or links should be provided. When possible, standardized format (SBML, CellML) should be used instead of scripts (e.g. MATLAB). Authors are strongly encouraged to follow the MIRIAM guidelines (see link list at top right) and deposit their model in a public database such as Biomodels (see link list at top right) or JWS Online (see link list at top right). If computer source code is provided with the paper, it should be deposited in a public repository or included in supplementary information.	NA

G- Dual use research of concern

22. Could your study fall under dual use research restrictions? Please check biosecurity documents (see link list at top right) and list of select agents and toxins (APHIS/CDC) (see link list at top right). According to our biosecurity guidelines, provide a statement only if it could.	NA
